# Supplementary material for: Mechanoelectronic stimulation of autologous extracellular vesicle biosynthesis implant for gut microbiota modulation
Source: Nat Commun. 2024 Apr 18;15:3343. doi: 10.1038/s41467-024-47710-w (PMC11026491; doi:10.1038/s41467-024-47710-w)
Supplement: Supplementary file 1 — Supplementary Information [file 41467_2024_47710_MOESM1_ESM.pdf]

Supplementary Information for

Mechanoelectronic stimulation of autologous extracellular vesicle biosynthesis implant for gut microbiota modulation

Shuangshuang Wan[1], Kepeng Wang[1], Peihong Huang[1], Xian Guo[1], Wurui Liu[1], Yaocheng Li[1], Jingjing Zhang[1], Zhiyang Li[2], Jiacheng Song[3], Wenjing Yang[1], Xianzheng Zhang[4], Xianguang Ding[1†], David Tai Leong[5†], Lianhui Wang[1†]

[1]State Key Laboratory of Organic Electronics and Information Displays & Jiangsu Key Laboratory for Biosensors, Institute of Advanced Materials (IAM), Nanjing University of Posts and Telecommunications, Nanjing 210023, China.
[2]Department of Clinical Laboratory Medicine, Nanjing Drum Tower Hospital, Nanjing University, Nanjing 210008, China.
[3]Department of Radiology, the First Affiliated Hospital of Nanjing Medical University, Nanjing 210023, China
[4]Key Laboratory of Biomedical Polymers of Ministry of Education & Department of Chemistry, Wuhan University, Wuhan 430072, China.
[5]Department of Chemical and Biomolecular Engineering, National University of Singapore, Singapore 117585, Singapore.

[*]Corresponding author. Email: iamxgding@njupt.edu.cn (X.G.D.); cheltwd@nus.edu.sg (D.T.L); iamlhwang@njupt.edu.cn (L.H.W.)

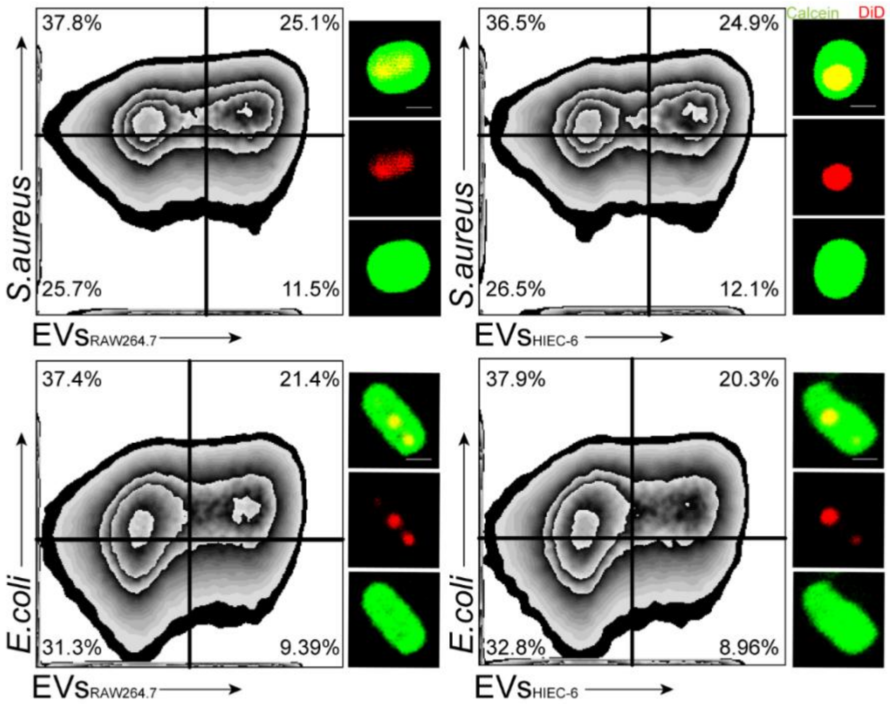

Supplementary Fig. 1. Two gut bacteria (*S.aureus* and *E. coli*) and two EVs from RAW264.7 ($EVs_{RAW264.7}$) and HIEC-6 cells (EVs_{HIEC-6}) were labeled with Calcein-AM (green fluorescent) and DiD (red fluorescent), respectively. After four hours of co-incubation with bacteria and EVs, bacterial uptake of EVs was analyzed by fluorescence colocalization. The experimental results indicated that intestinal bacteria were able to phagocytize EVs originating from host cells, facilitating the transmission of information between two different species. Scale bar: 1μm.

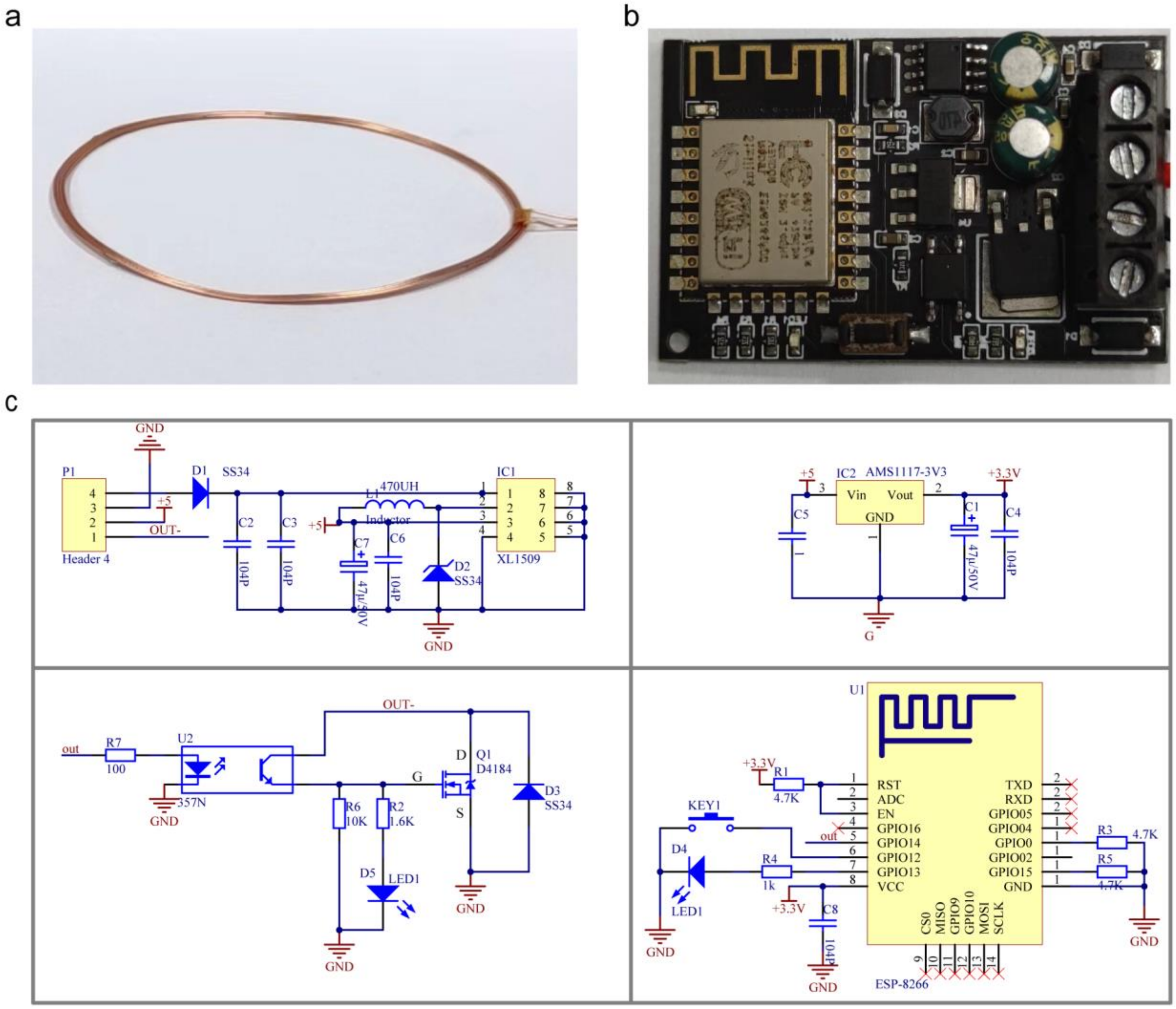

Supplementary Fig. 2. Layout of the wireless control module. (a) The image of the designed wireless induction coil. (b) The chip of the wireless control module. (c) Layout and component information of wireless control module.

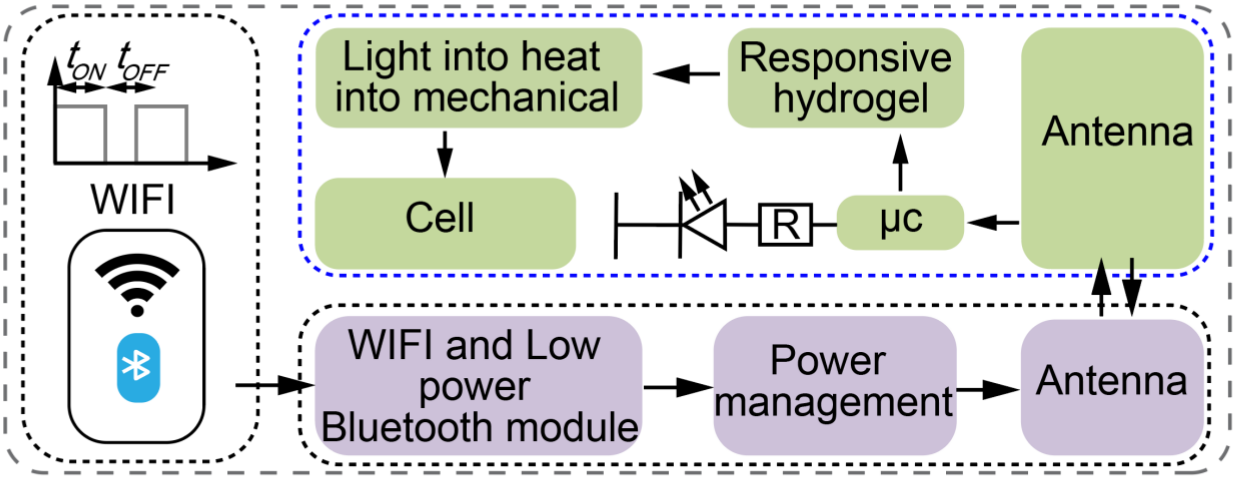

Supplementary Fig. 3. System block diagram of the entire iMASSAGE system design featuring wireless interconnected electronic devices. To connect the iMASSAGE operation to a user interface, the iMASSAGE system incorporates a wireless power management module to transfer the electronic signal wirelessly via resonant inductive coupling. This enables iMASSAGE system to exert dynamical mechanical stimulus on embedded cells in a wireless and programmable manner through electronic devices.

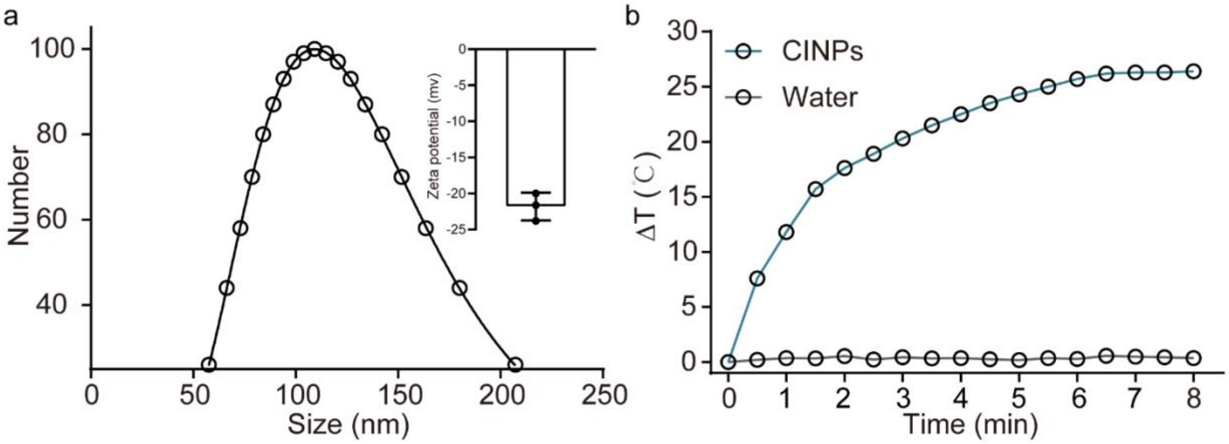

Supplementary Fig. 4. (a) The size and zeta potential (inset) of CINPs by DLS. (b) Temperature change of CINPs with varied irradiation time. It shows that CINPs could be employed as good photothermal agents for photothermal energy conversion. Source data are provided as a Source Data file.

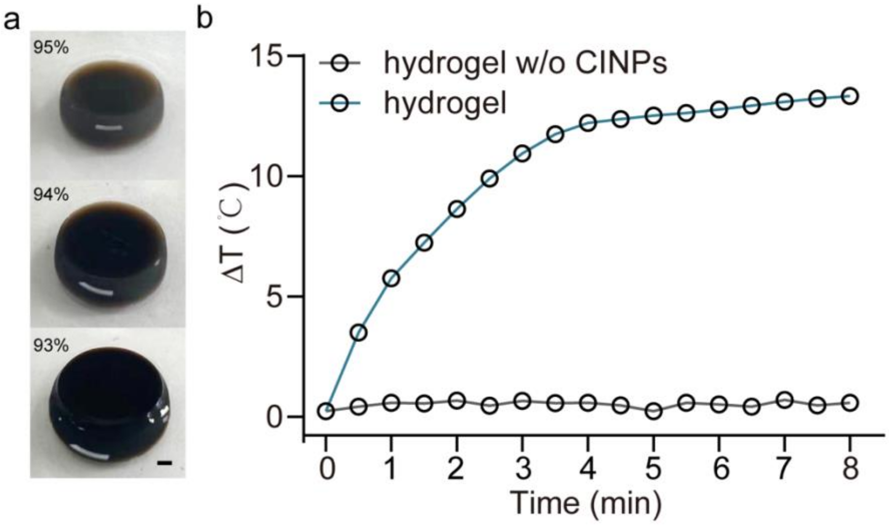

Supplementary Fig. 5. (a) The images of synthesized hydrogels with different precursor N-isopropyl acrylamide ratios. Scale bar: 1mm. (b) Temperature change of the hydrogel doped with or without CINPs over radiation time (1 W). Source data are provided as a Source Data file.

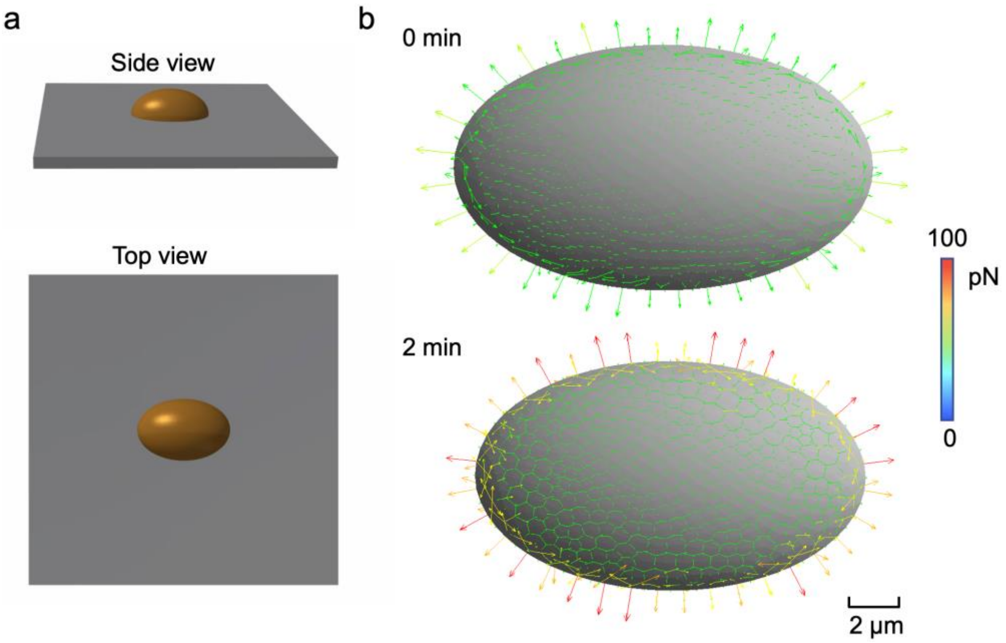

Supplementary Fig. 6. Computed stress distributions for a strain cell on the contractible hydrogel. (a) Scheme of a cell adhered on hydrogel surface. The cell was conceptually represented as an ellipsoidal shape on the hydrogel, featuring dimensions of 2 μm in height, 15 μm in length and 8 μm in width. (b) Top view of the force vector and density of cells embedded in contracted hydrogel at different time points.

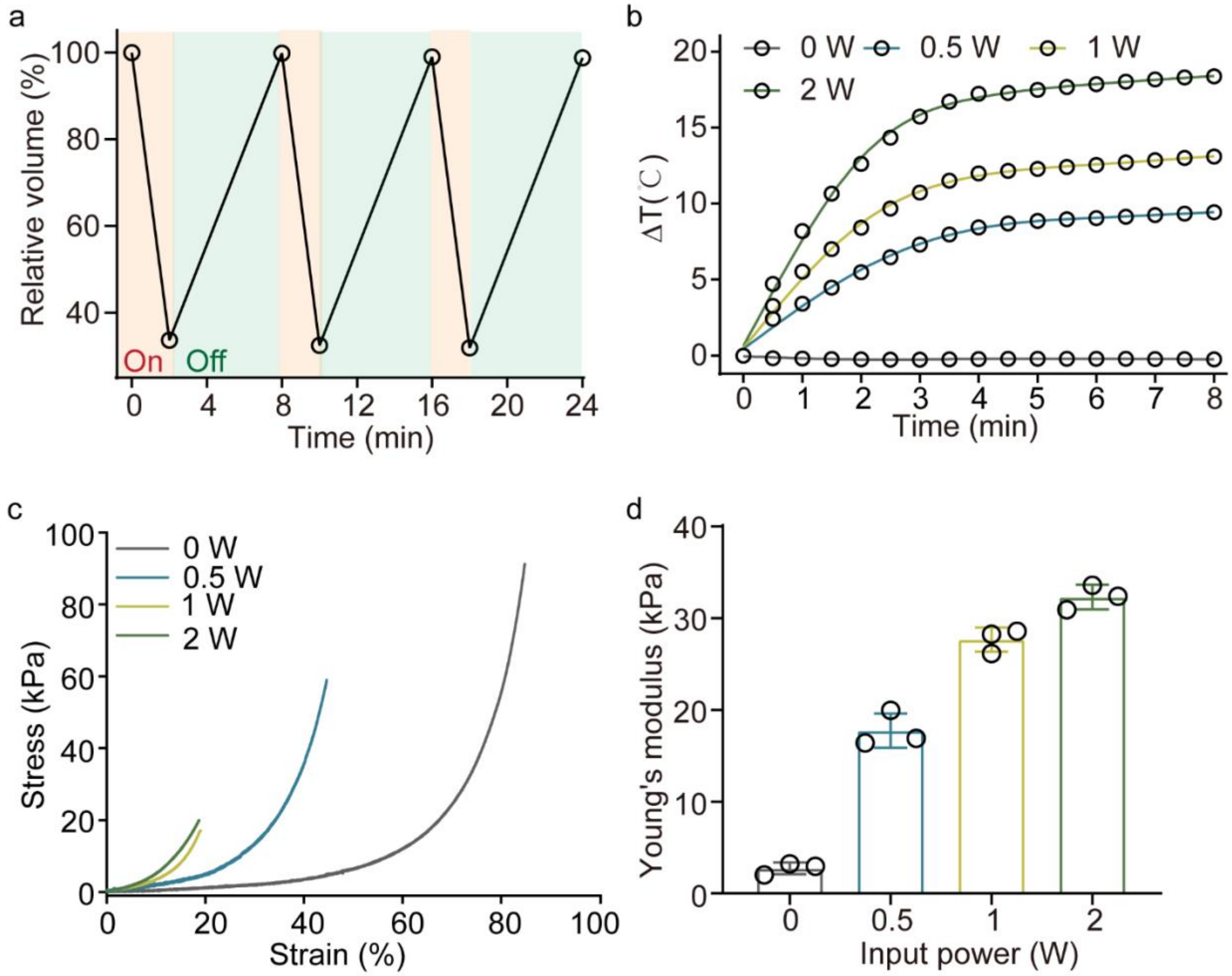

Supplementary Fig. 7. Characterization of mechanical properties of the hydrogel-encapsulated device. (a) Volumetric change of the device under cyclic wireless stimulation (1W). (b) The temperature change of the device at different wireless input powers. (c) Stress-strain curves of the device after two minutes of varying wireless input power. (d) Young's modulus of iMASSAGE system in the maximum shrinkage state at different wireless input powers (n=3 independent experiments). All data are presented as mean ± s.d.. Source data are provided as a Source Data file.

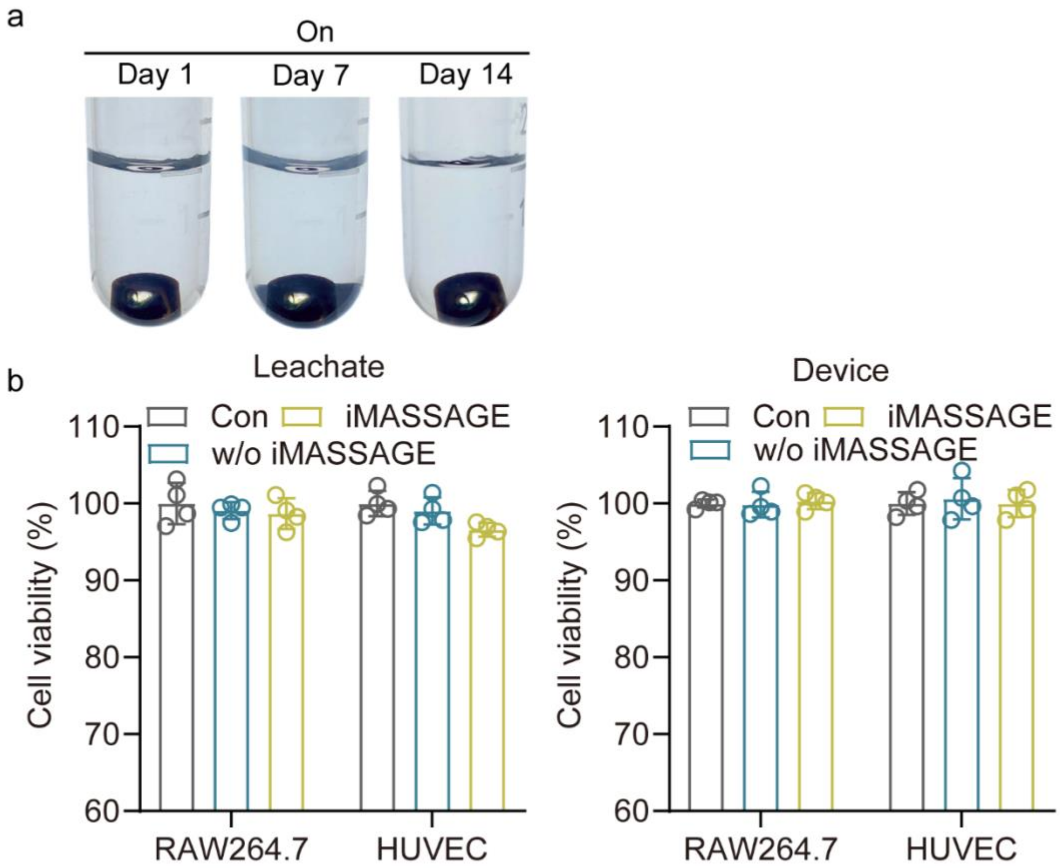

Supplementary Fig. 8. Stability and biocompatibility of iMASSAGE device.
(a) Images of the device immersed in PBS buffer on different days, suggestive that the device remained functional over 14 days. (b) The cytotoxicity of the device and its leachate on RAW264.7 and HUVEC cells by incubating them separately for 5 days (iMASSAGE means the device was subjected to wireless stimulation. 1 W, 1T) (n=3 independent experiments). All data are presented as mean ± s.d.. Source data are provided as a Source Data file.

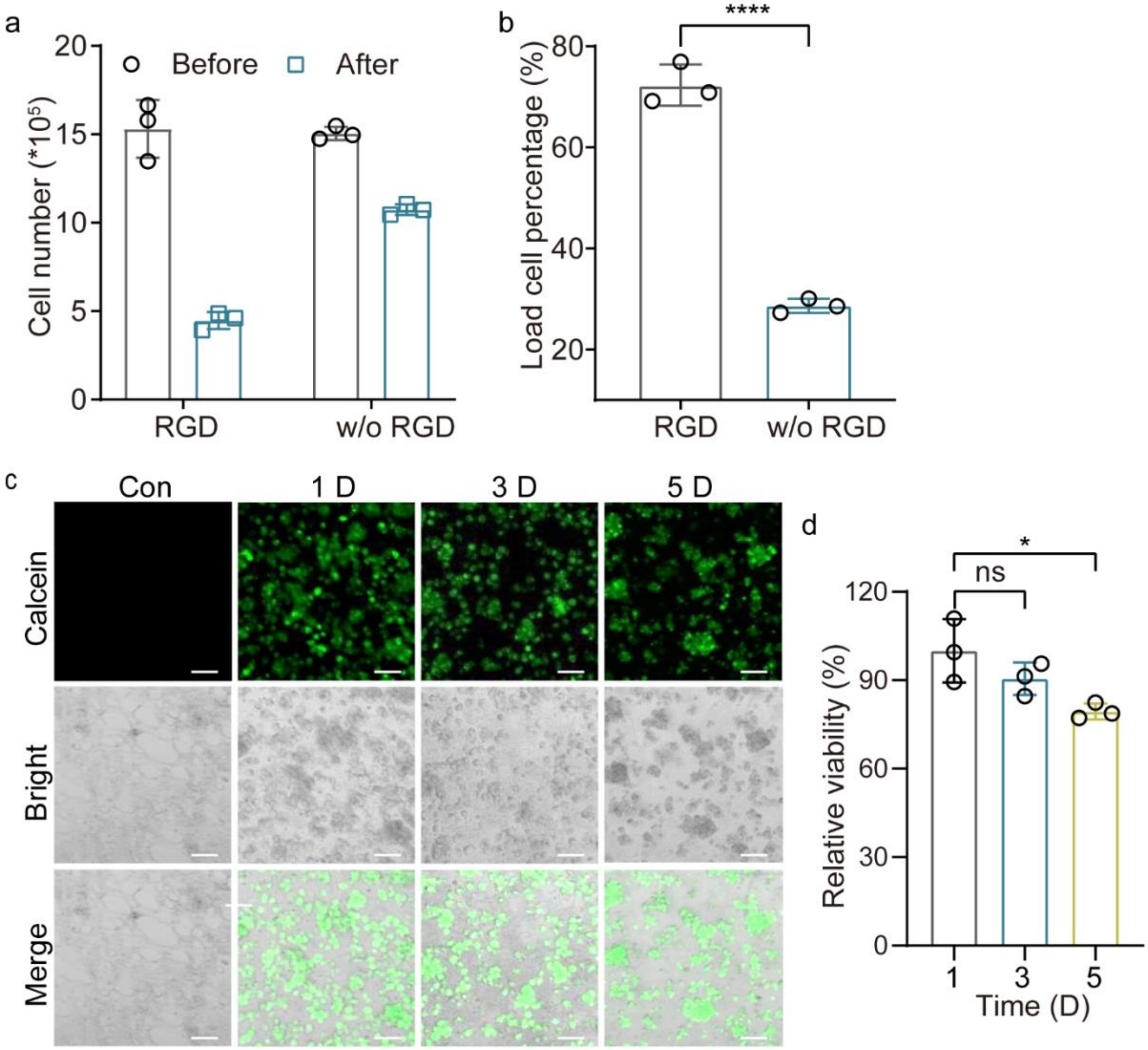

Supplementary Fig. 9. Loading of cell by iMASSAGE device. (a) Cell numbers in the medium before and after cell loading in the device with or without RGD modification (n=3 independent experiments). (b) Percentage of cells loaded into device with or without RGD modification (n=3 independent experiments). (c) Confocal images of live cells within the device stained with Calcein-AM (green) at different incubation days and (d) the corresponding cell viability (with macrophages embedded as model cells) (n=3 independent experiments). Scale bar: 50 μm. The significant differences were calculated based on a two-tailed Student's t-test. *$p < 0.05$, ns refer to no significant. All data are presented as mean ± s.d.. Source data are provided as a Source Data file.

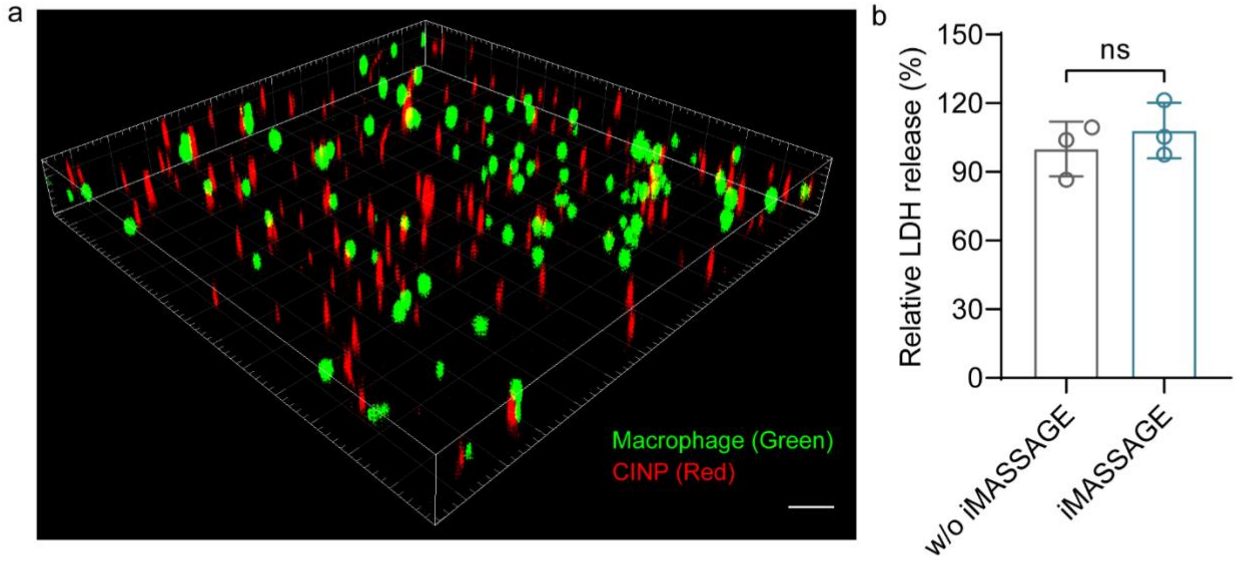

Supplementary Fig. 10. (a) Representative 3D confocal image of iMASSAGE hydrogel showing the distribution of macrophages (green labeled) and CINPs (red labeled). Scale bar: 50 µm. (b) LDH release of cells embedded in the device with/without pulsed wireless stimulation (n=3 independent experiments). The significant differences were calculated based on a two-tailed Student's t-test. ns refer to no significant. All data are presented as mean ± s.d.. Source data are provided as a Source Data file.

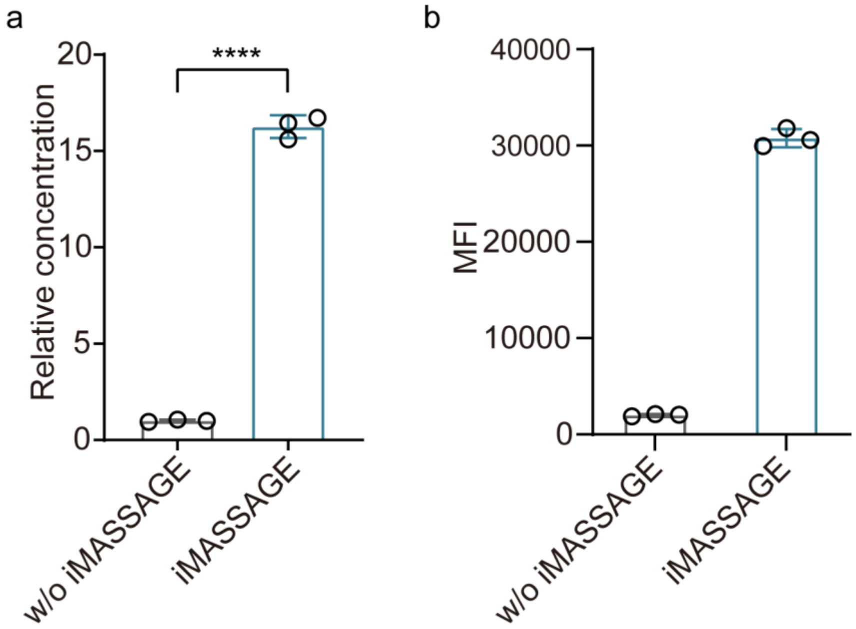

Supplementary Fig. 11. iMASSAGE-induced EV generation. (a) Relative concentration of EVs detected by NTA (n=3 independent experiments). (b) Mean fluorescence intensity by flow cytometry after iMASSAGE stimulation (n=3 independent experiments). The significant differences were calculated based on a two-tailed Student's t-test. ****p < 0.0001. All data are presented as mean ± s.d.. Source data are provided as a Source Data file.

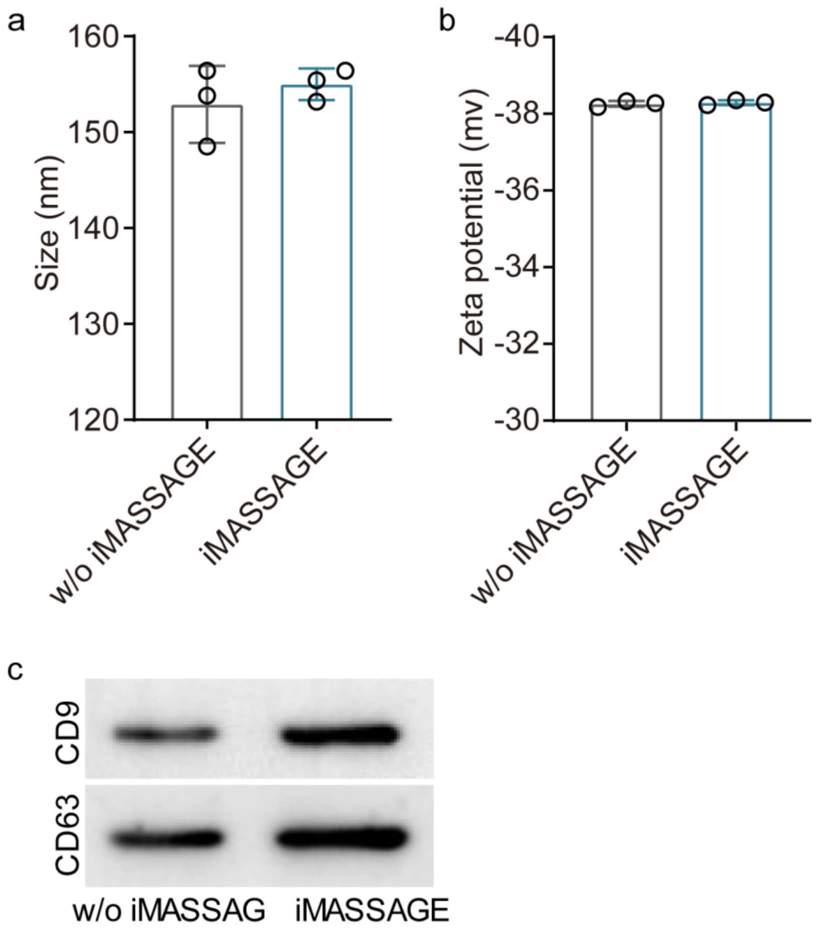

Supplementary Fig. 12. Physical and compositional characterization of EVs by iMASSAGE. (a) Size and (b) zeta potential of EVs detected by DLS (n=3 independent experiments). (c) Bands of characteristic proteins of EVs by WB with/without iMASSAGE stimulation. All data are presented as mean ± s.d.. Source data are provided as a Source Data file.

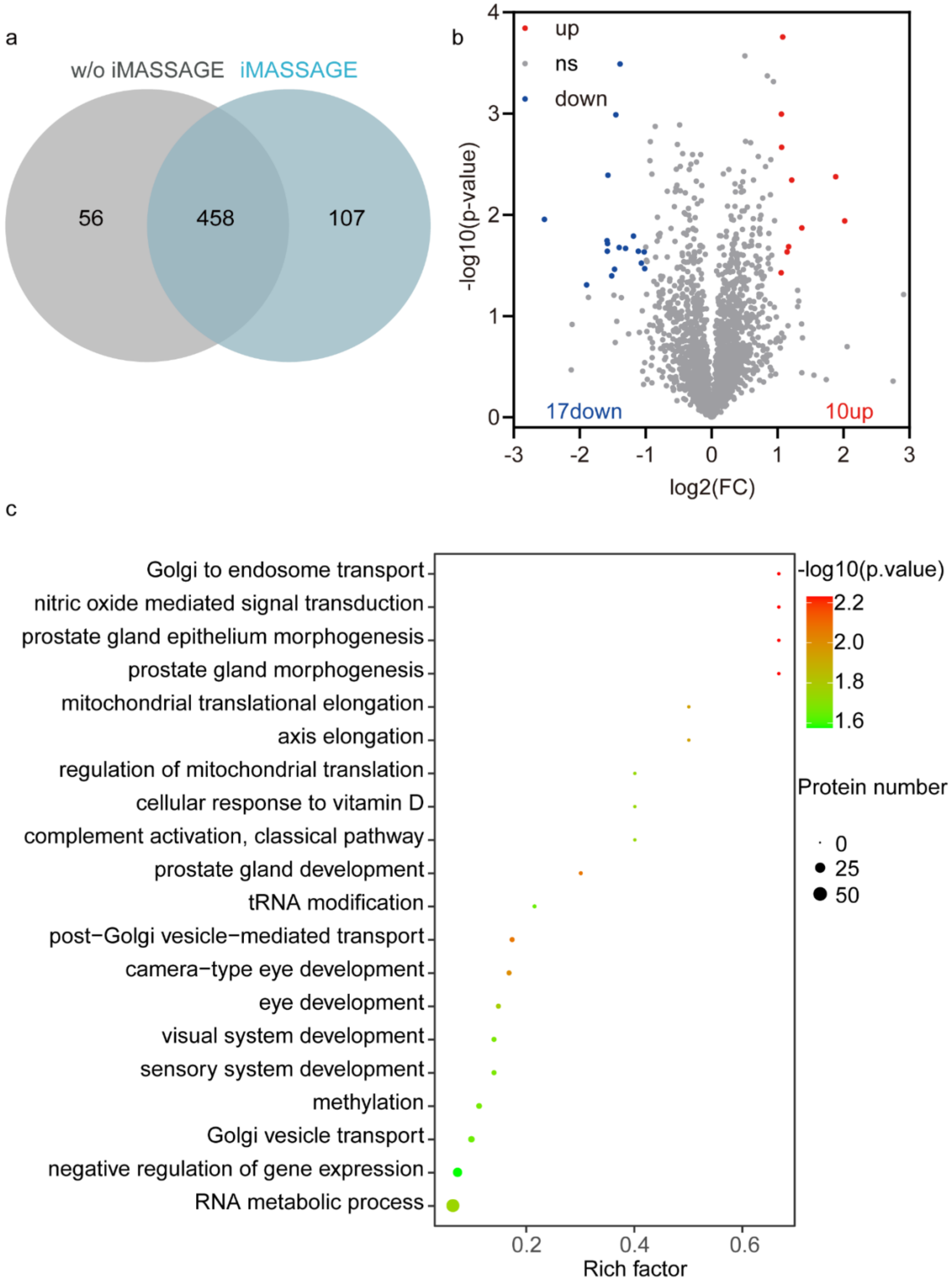
a
w/o iMASSAGE
iMASSAGE
56
458
107
b
up
ns
down
-log10(p-value)
17down
10up
log2(FC)
c
Golgi to endosome transport
nitric oxide mediated signal transduction
prostate gland epithelium morphogenesis
prostate gland morphogenesis
mitochondrial translational elongation
axis elongation
regulation of mitochondrial translation
cellular response to vitamin D
complement activation, classical pathway
prostate gland development
tRNA modification
post−Golgi vesicle−mediated transport
camera−type eye development
eye development
visual system development
sensory system development
methylation
Golgi vesicle transport
negative regulation of gene expression
RNA metabolic process
-log10(p.value)
2.2
2.0
1.8
1.6
Protein number
0
25
50
0.2
0.4
0.6
Rich factor

Supplementary Fig. 13. Proteomic analysis of EVs with or without iMASSAGE treatment. (a) Venn diagram showing identified protein overlap between iMASSAGE and w/o iMASSAGE groups. (b) Volcano plot showing differences in total identified protein between iMASSAGE and w/o iMASSAGE groups. Upregulated protein (red, FC > 2 and P < 0.05) and downregulated protein (blue, FC < 0.5 and P < 0.05). (c) Gene Ontology (GO) enrichment analysis of biological process (BP) using Fisher's Exact Test.

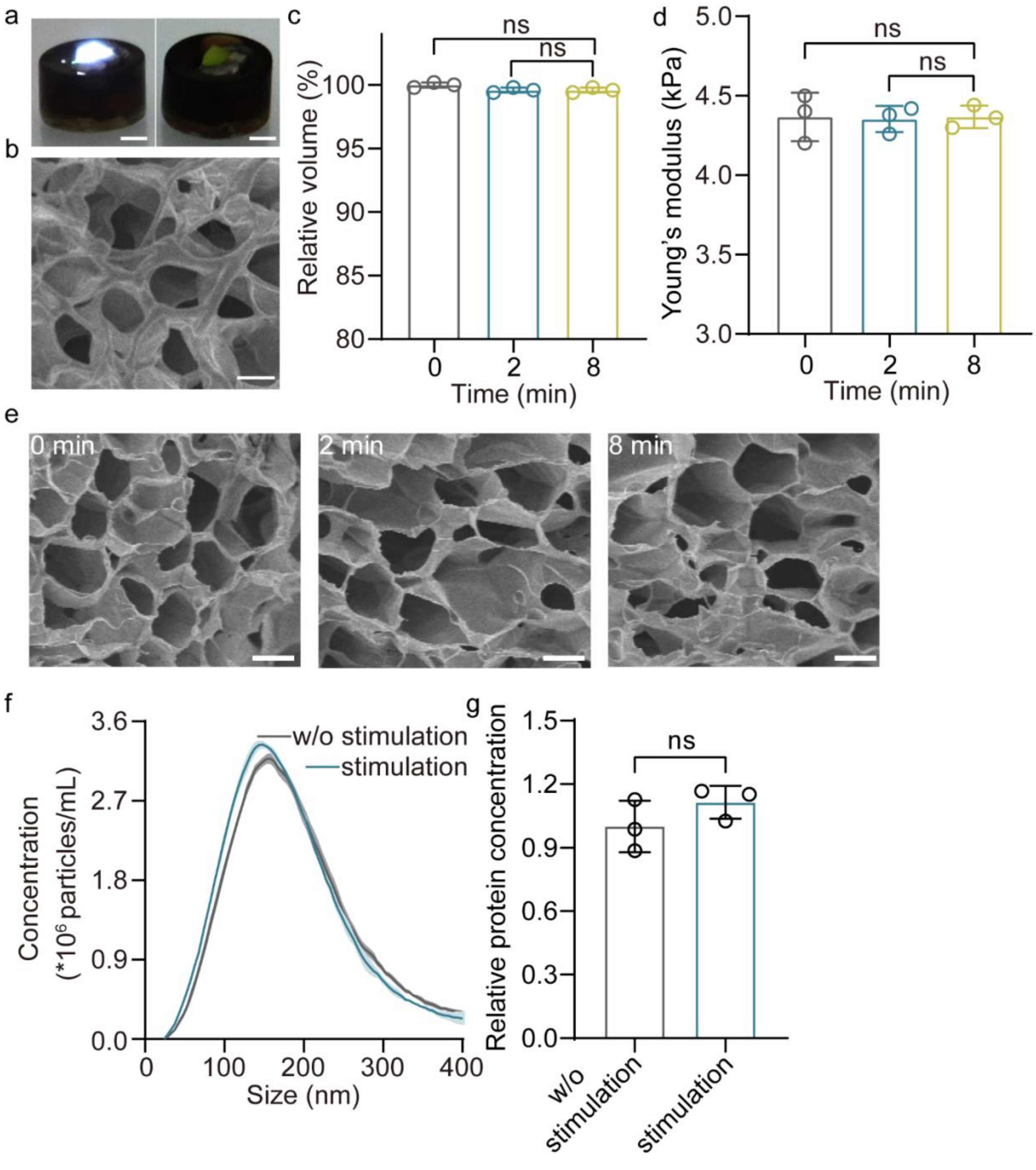

Supplementary Fig. 14. Construction and performance evaluation of PAM device. (a) The image of PAM device activated by wireless. Scale bar: 1mm. (b) Representative SEM image of pore size of PAM device. Scale bar: 50 μm. (c) The relative volume and (d) Young's modulus of PAM device in its original state (0 min), the wireless-induced contraction state (2 min) and relaxation state (8 min) (n=3 independent experiments). (e) Representative SEM images of the internal aperture of PAM device at 0, 2 and 8 min. Scale bar: 50 μm. PAM-produced EV concentration detected by (f) NTA and (g) BCA kit with or without stimulation

(n=3 independent experiments). The significant differences were calculated based on a two-tailed Student's t-test. ns refers to no significance. All data are presented as mean ± s.d.. Source data are provided as a Source Data file.

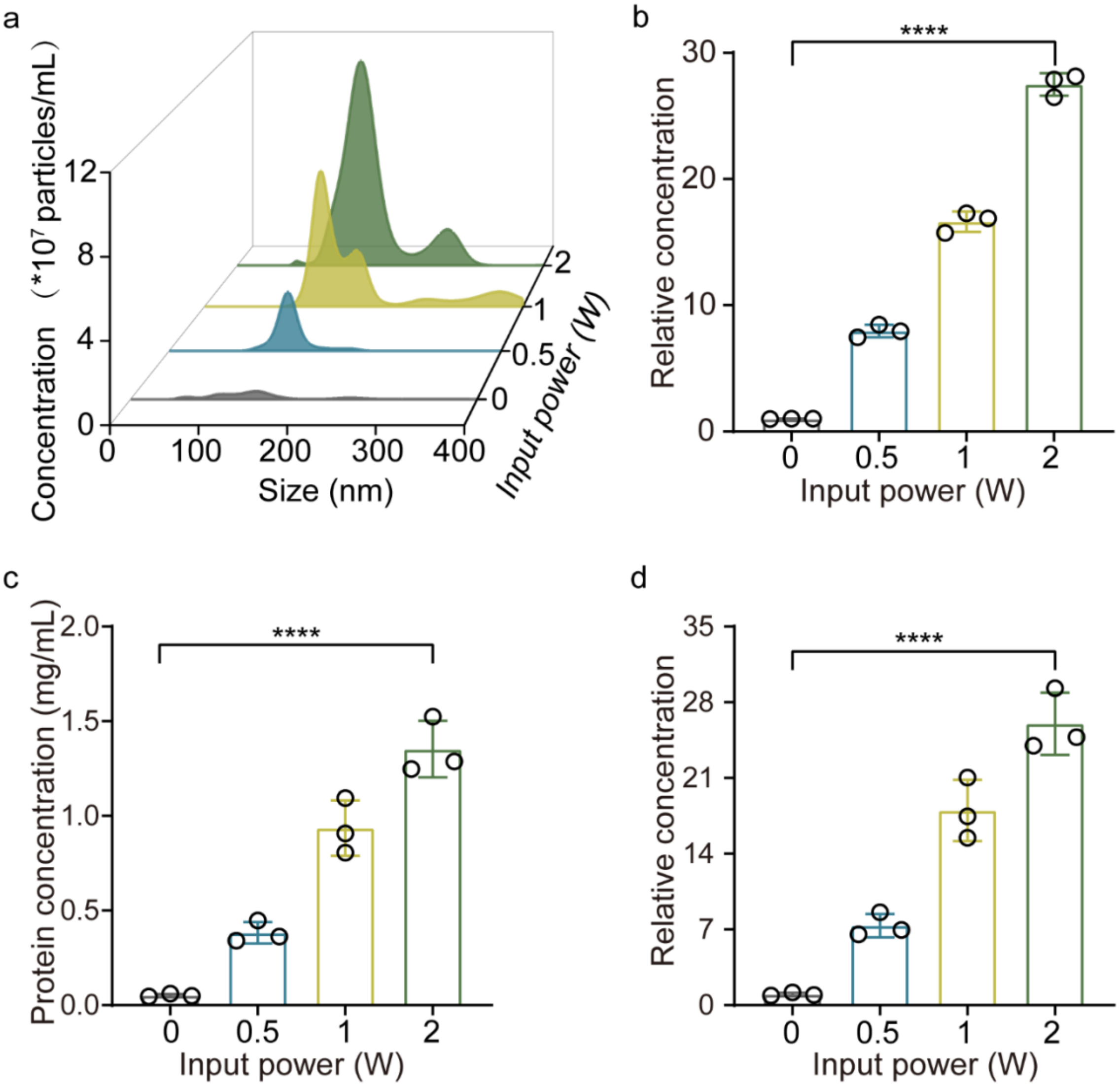

Supplementary Fig. 15. iMASSAGE-mediated EVs release as a function of input power. (a) The concentration of iMASSAGE-released EVs and (b) relative concentration at different input powers quantified with NTA (n=3 independent experiments). (c) Protein concentration and (d) relative concentration of iMASSAGE-released EVs at different input powers detected by BCA kit (n=3 independent experiments). The significant differences were calculated based on a two-tailed Student's t-test. ****$p < 0.0001$. All data are presented as mean ± s.d.. Source data are provided as a Source Data file.

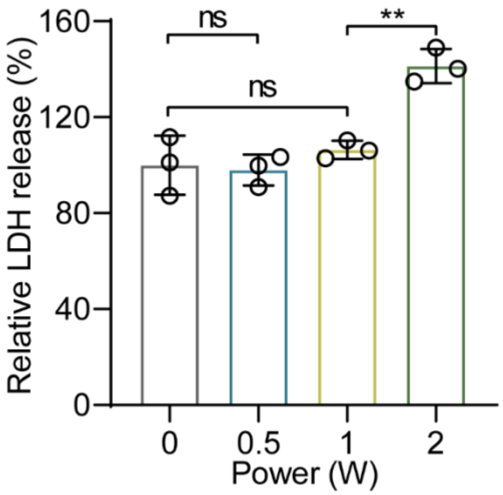

Supplementary Fig. 16. The viability of colonized cells in iMASSAGE treated with different input power by LDH release (n=3 independent experiments). The significant differences were calculated based on a two-tailed Student's t-test. **$p < 0.01$, ns refers to no significant. All data are presented as mean ± s.d.. Source data are provided as a Source Data file.

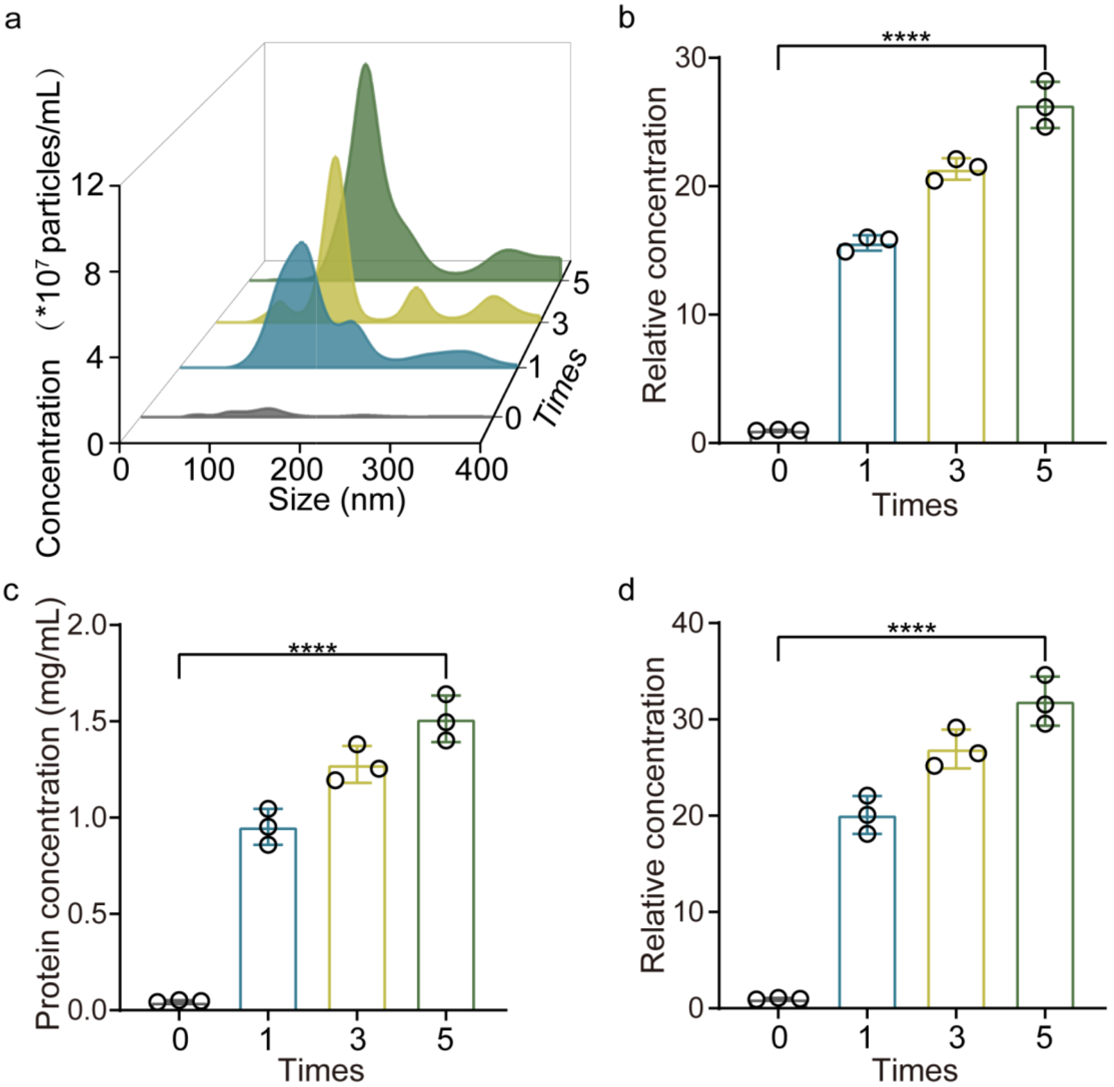

Supplementary Fig. 17. iMASSAGE-mediated EV release as a function of iMASSAGE times. (a) iMASSAGE-released EV concentration and (b) relative concentration at different iMASSAGE times using NTA (n=3 independent experiments). (c) Protein concentration and (d) relative concentration of iMASSAGE-released EVs at different iMASSAGE times detected by BCA kit (n=3 independent experiments). The significant differences were calculated based on a two-tailed Student's t-test. ****$p < 0.0001$. All data are presented as mean ± s.d.. Source data are provided as a Source Data file.

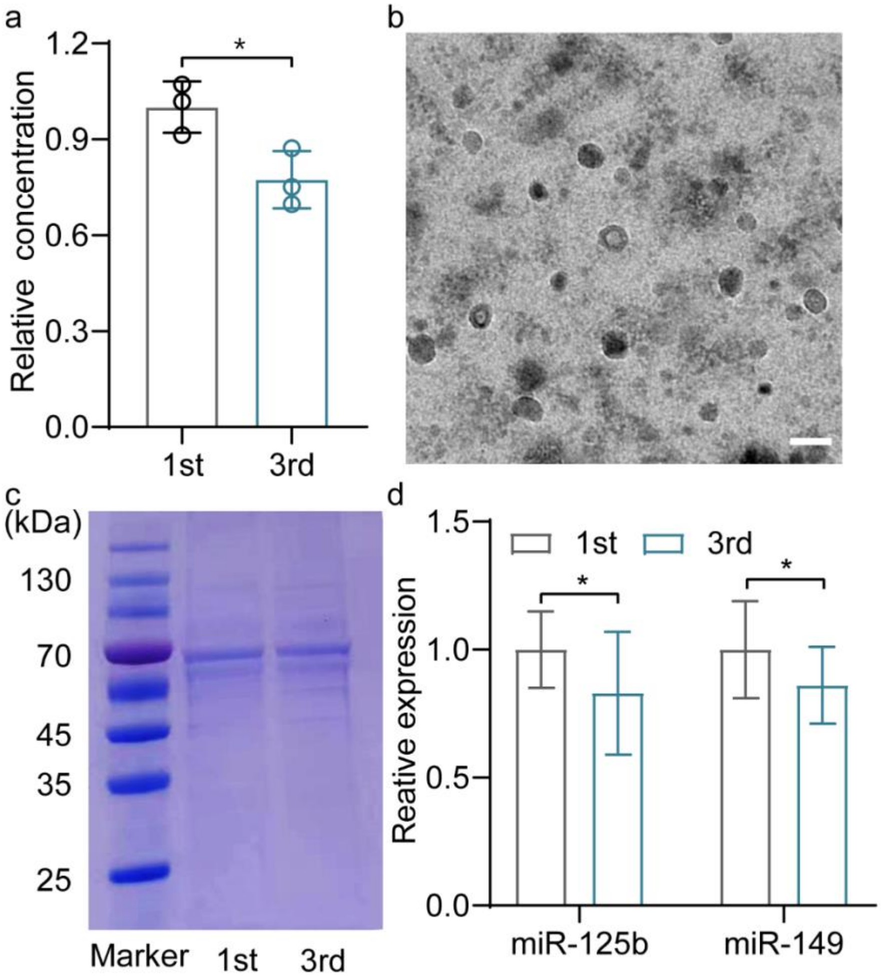

Supplementary Fig. 18. Differences in EVs produced by iMASSAGE device between single- and multiple-pulse wireless stimulation. (a) Concentration of EVs released from iMASSAGE with first stimulation and third stimulation (n=3 independent experiments). (b) TEM image of iMASSAGE-released EVs by the third stimulation. Scale bar: 200 nm. (c) SDS-PAGE protein analysis of EV from iMASSAGE with first cycle stimulation and third cycle stimulation. (d) The relative expression of miRNA-125b and miR-149 from iMASSAGE-released EVs with first stimulation and third stimulation (n=3 independent experiments). The significant differences were calculated based on a two-tailed Student's t-test. $^{**}p < 0.01$, ns refers to no significant. All data are presented as mean ± s.d.. Source data are provided as a Source Data file.

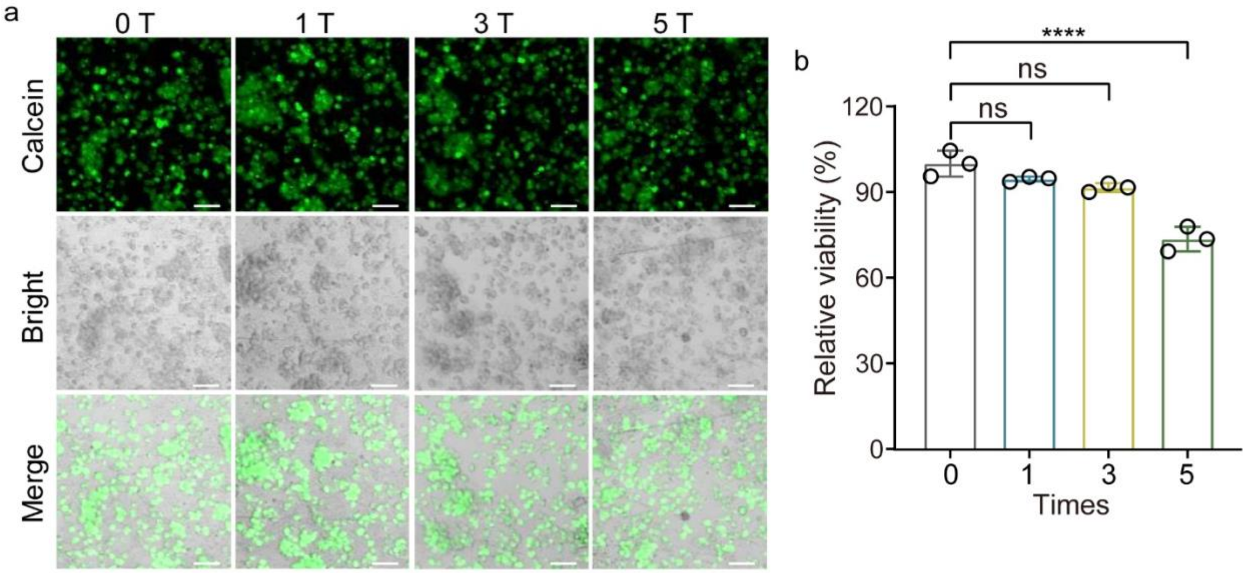

Supplementary Fig. 19. The viability of colonized cells before and after iMASSAGE stimulation (1 W, different times). (a) Representative live cell staining images with Calcein-AM (green) observed by confocal and (b) the corresponding viability of cells (n=3 independent experiments). Scale bar: 50 µm. The significant differences were calculated based on a two-tailed Student's t-test. ****P < 0.0001, ns refers to no significant. All data are presented as mean ± s.d.. Source data are provided as a Source Data file.

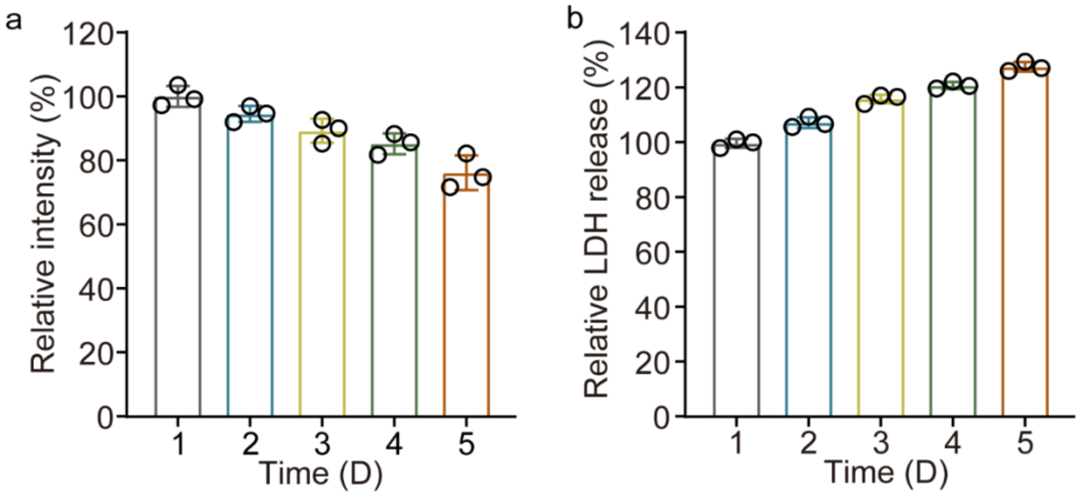

Supplementary Fig. 20. The cell viability after intermittent iMASSAGE stimulation. (a) The semi-quantitative data of Calcein-AM fluorescence (n=3 independent experiments). (b) The viability of cells residing in the device was determined by detecting LDH release. The iMASSAGE stimulations were applied on the first, third and fifth days (n=3 independent experiments). All data are presented as mean ± s.d.. Source data are provided as a Source Data file.

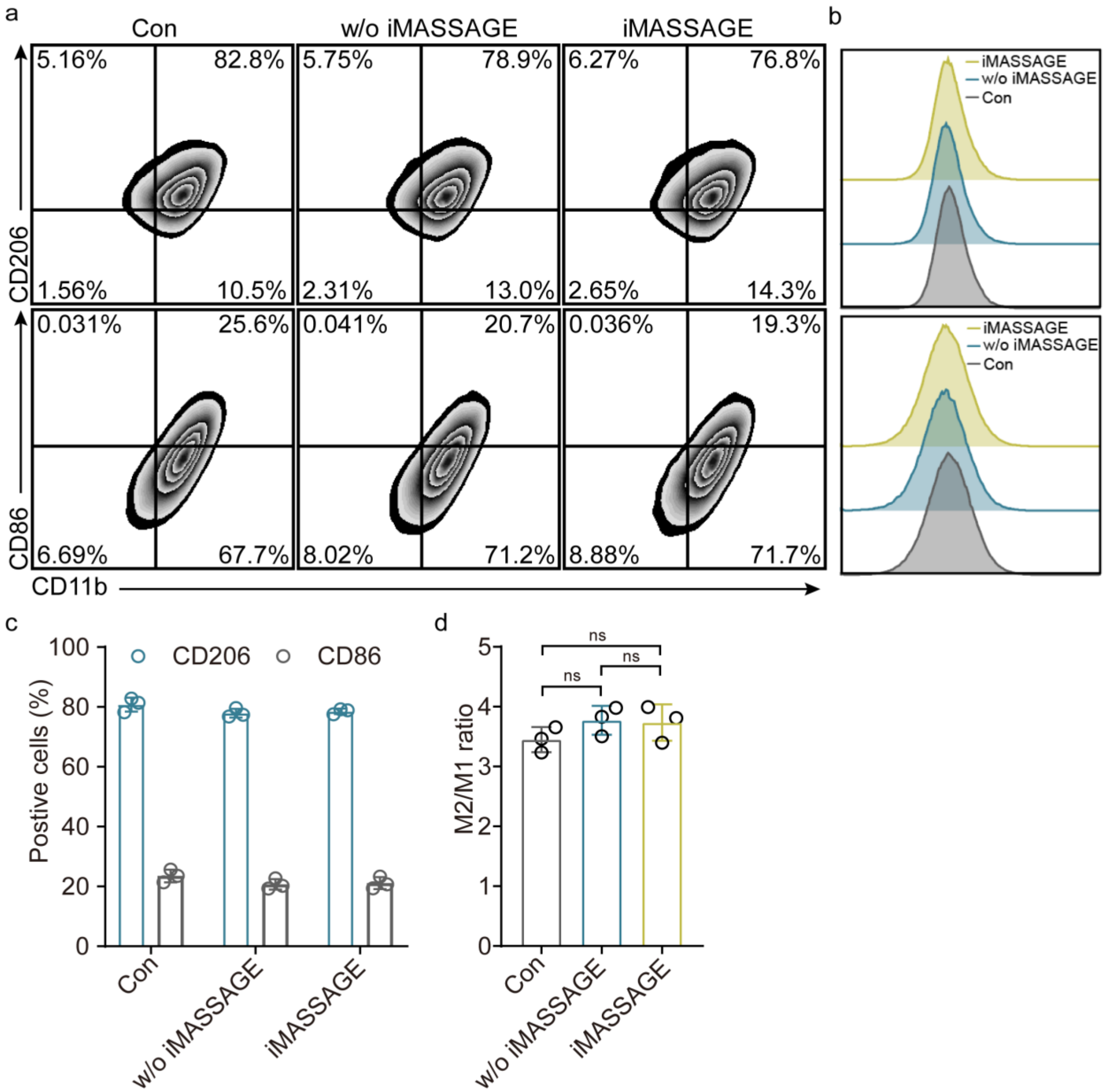

Supplementary Fig. 21. (a) The percentage of M2 ($CD11b^{+}CD206^{+}$) and M1 ($CD11b^{+}CD86^{+}$) macrophages after different treatments. (b) The fluorescence intensity of CD206 and CD86 antibodies. (c) Corresponding statistical percentage of positive cells and (d) the M2/M1 macrophage ratio after different treatments (n=3 independent experiments). The significant differences were calculated based on two-tailed Student's t-test. ns refers to no significant. All data are presented as mean ± s.d.. Source data are provided as a Source Data file.

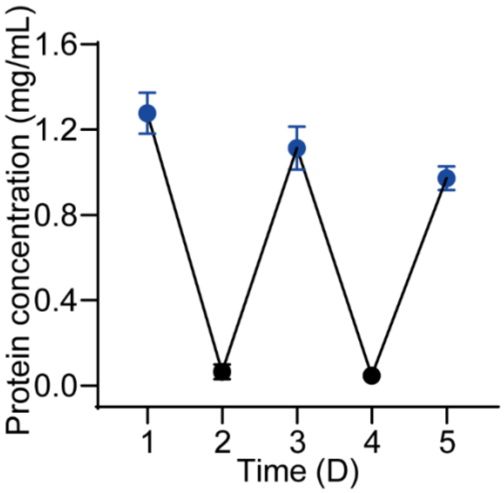

Supplementary Fig. 22. The protein concentration of collected EVs detected by BCA (n=3 independent experiments). Source data are provided as a Source Data file. iMASSAGE stimulation (3 T and 1 W, blue dot) was employed on days 1, 3 and 5, without stimulation on days 2 and 5 (black dot).

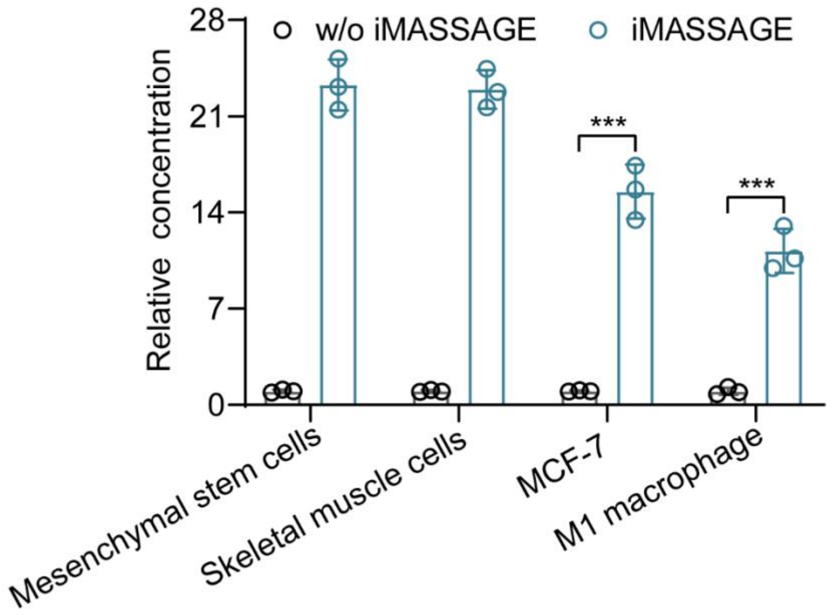

Supplementary Fig. 23. The relative protein concentration of collected EVs from different cell-resided iMASSAGE systems (1 W, 3 T) (n=3 independent experiments). All data are presented as mean ± s.d.. The significant differences were calculated based on a two-tailed Student's t-test. ***$p < 0.001$. Source data are provided as a Source Data file.

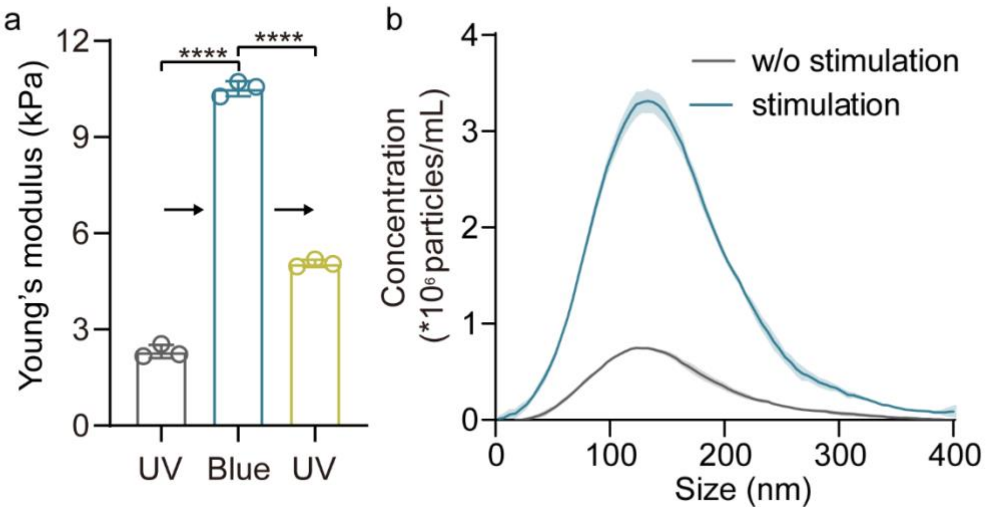

Supplementary Fig. 24. (a) Young's modulus of AZO-PA hydrogel under irradiation. AZO-PA hydrogel was exposed to UV light (365 nm) for 10 min, blue light (490 nm) for 2 h and then UV light (365 nm) for 10 min. (b) The concentration of EVs by NTA with or without stimulation (photo stimulation: 10 min of UV, 2 h of blue and 10 min of UV) (n=3 independent experiments). All data are presented as mean ± s.d.. The significant differences were calculated based on a two-tailed Student's t-test. ****$p < 0.0001$. Source data are provided as a Source Data file.

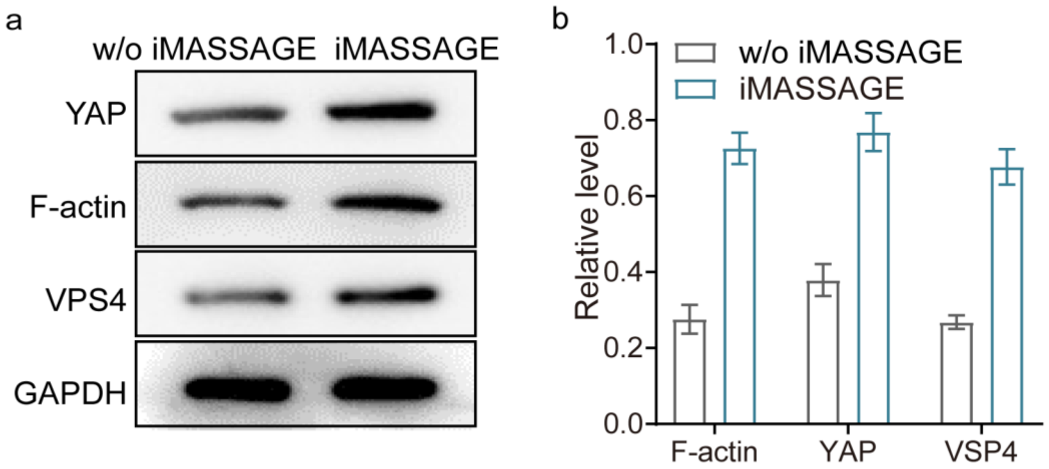

Supplementary Fig. 25. Expression of EV generation-related proteins in the colonized cells under iMASSAGE treatment (1 W, 3 T). (a) Protein bands of colonized cells and (b) the corresponding relative protein expression treated with or without iMASSAGE by western blot (n=3 independent experiments). Source data are provided as a Source Data file.

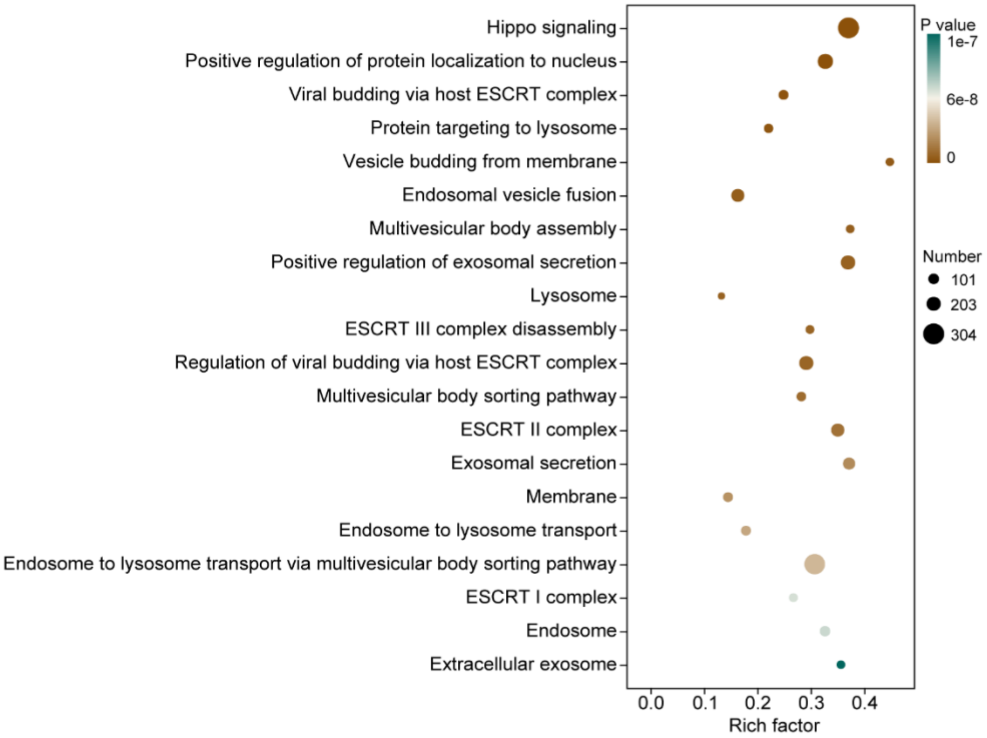

Supplementary Fig. 26. The Gene Ontology (GO) analysis of the top 20 GO terms.

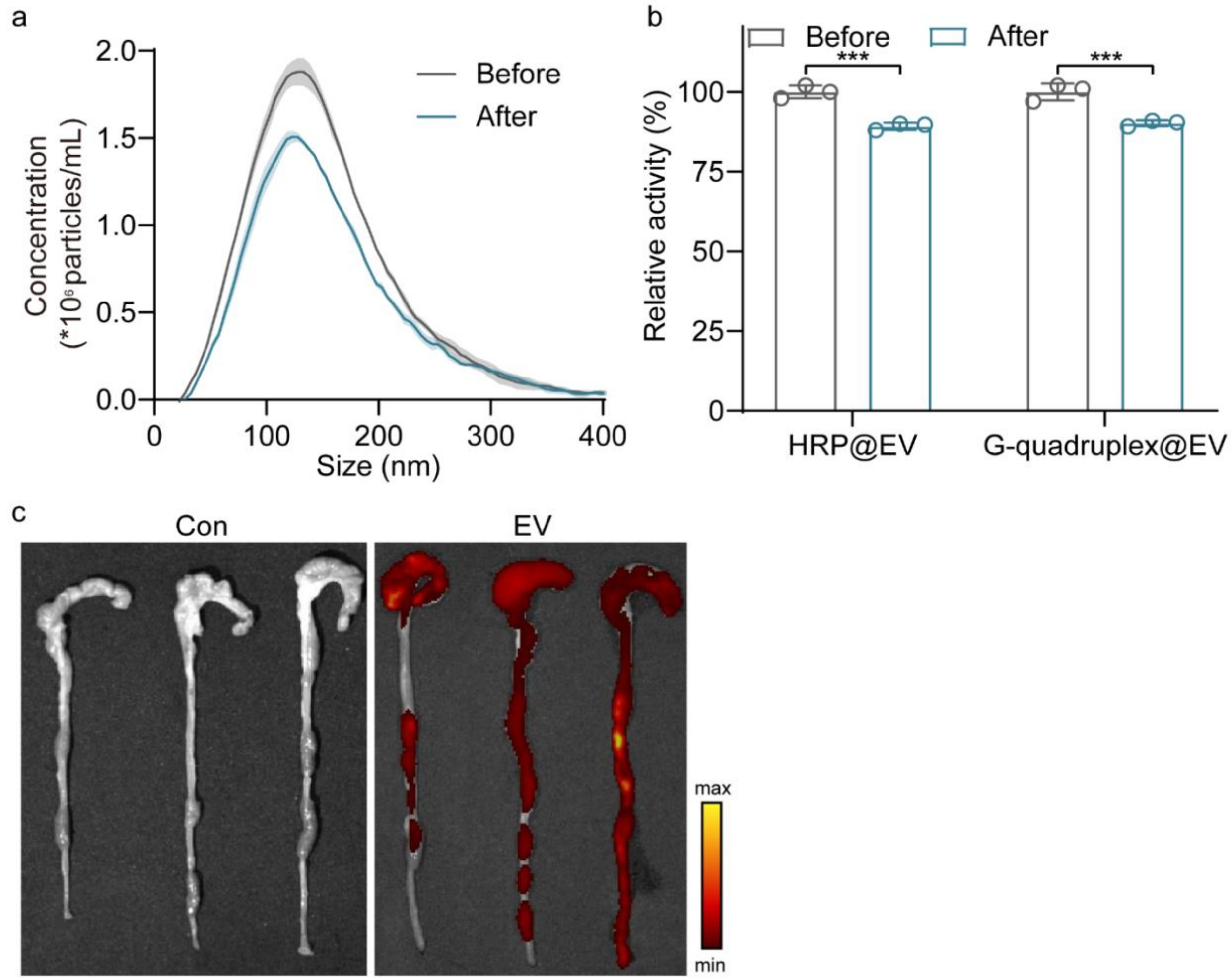

Supplementary Fig. 27. Intestinal lumen colonization of EVs by oral administration. (a) The concentration of EVs before and after immersion in SGF detected by NTA test. (b) The activity of HRP and G-quadruplex in EV before and after immersion in SGF detected by kit (n=3 independent experiments). (c) The iMASSAGE-generated EVs labeled with Did were imaged after oral administration to healthy mice. Control group (Con) was healthy mice without any treatment. All data are presented as mean ± s.d.. The significant differences were calculated based on a two-tailed Student's t-test. ns refers to no significance. ***$p < 0.001$. Source data are provided as a Source Data file.

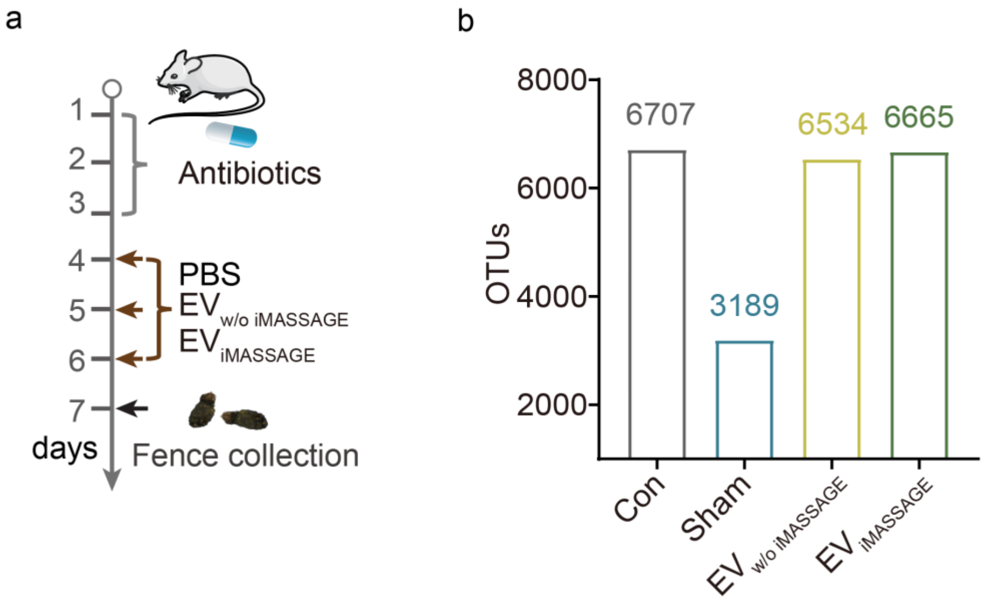

Supplementary Fig. 28. Different treatments for gut microbial modulation. (a) Timeline of EVs-medicated gut microbial regulation using iMASSAGE technology. Disturbed gut microbiome was simulated by feeding water-containing antibiotics (metronidazole, neomycin, vancomycin, and ampicillin) to healthy mice (n=5) for three days. And then, the iMASSAGE system-generated EVs ($EV_{iMASSAGE}$) and commonly cultured strategy-derived EVs (EV_{common}) (macrophages generated, with equal amounts of 1.0 $\times 10^{10}$ EV particles/g) were orally administered to the mice. Mouse feces were collected for microbiota analysis by 16s RNA sequencing on the seventh day. (b) The representative total number of OTUs from Venn diagram by statistics.

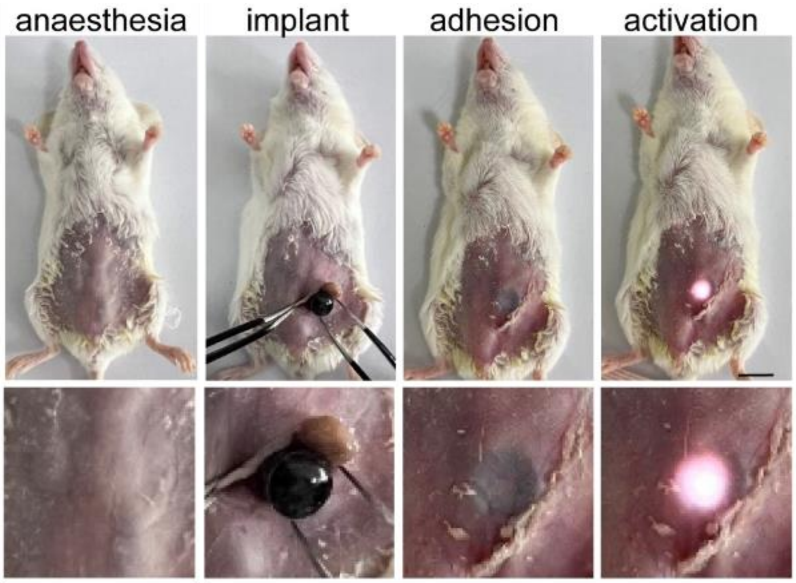

Supplementary Fig. 29. Images of surgically implanted devices. After being anesthetized with isoflurane, the mice were surgically laparotomy and implanted with iMASSAGE device. And then, the wound was sealed using bioglue. The device was successfully activated *in vivo* under wireless control. Scale bar: 5 mm.

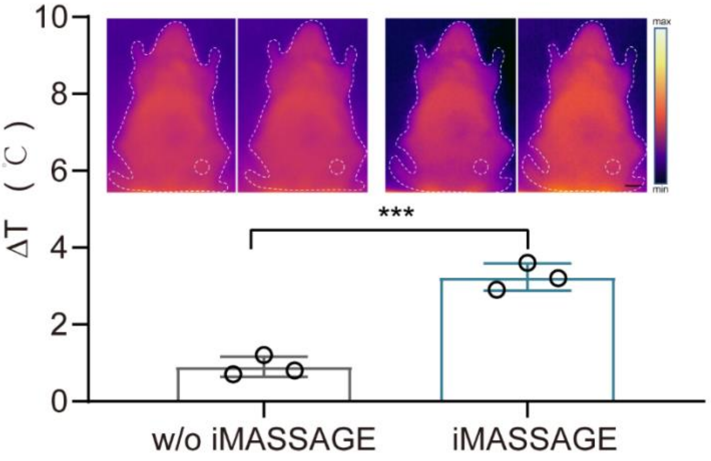

Supplementary Fig. 30. ***In vivo*** **photothermal imaging.** The images (up) and temperature difference (ΔT, down) of photothermal imaging of implanted devices in mice before and after pulse wireless control (n=3 mice per group). The white circle represents the position of the implanted device. Scale bar: 5 mm. All data are presented as mean ± s.d.. The significant differences were calculated based on a two-tailed Student's t-test. ***p < 0.001. Source data are provided as a Source Data file.

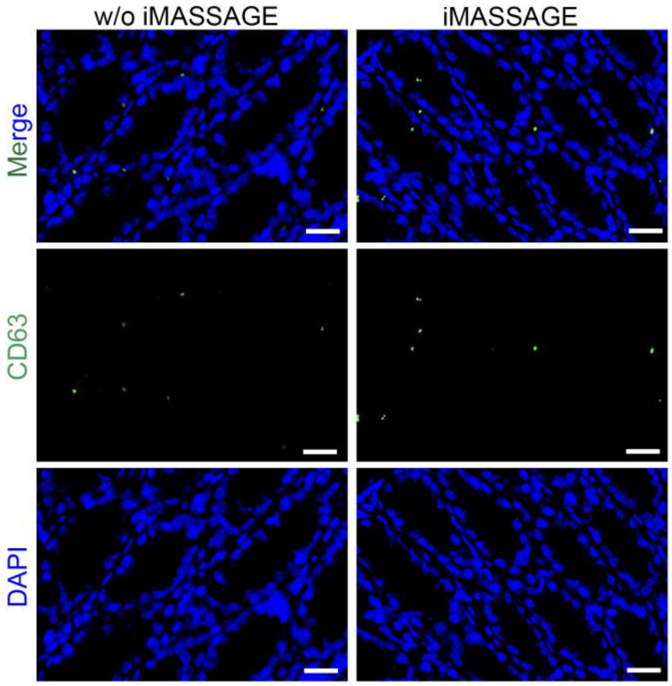

Supplementary Fig. 31. Specific anti-CD63 staining of tissues near the implant without cell loading. Scale bar: 20 μm.

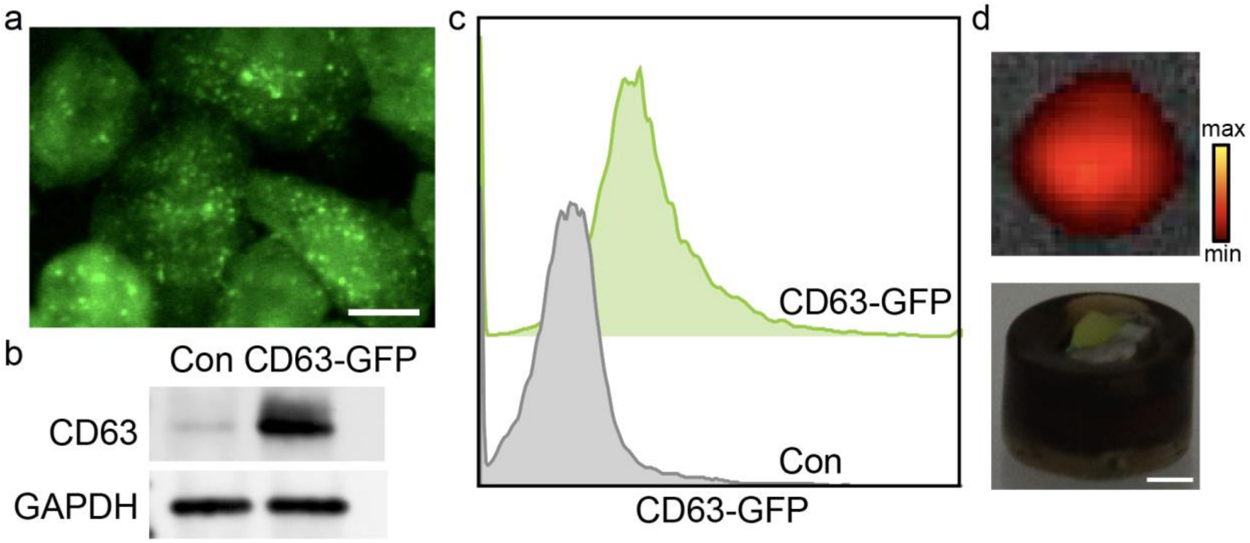

Supplementary Fig. 32. Construction of stably transfected macrophages with CD63-GFP gene for EV monitoring in vivo by iMASSAGE system. Transfected M2 macrophages expressing CD63-GFP proteins visualized by (a) confocal microscopy and detected by (b) western blot. Scale bar: 10 µm. Con refers to normal cell and CD63-GFP refers to M2 macrophages expressing CD63-GFP proteins. (c) Detection of transfected macrophages-produced EV contained CD63-GFP proteins by flow cytometry. (d) The image of iMASSAGE device loaded transfected M2 macrophages and imaged by IVIS imaging system.

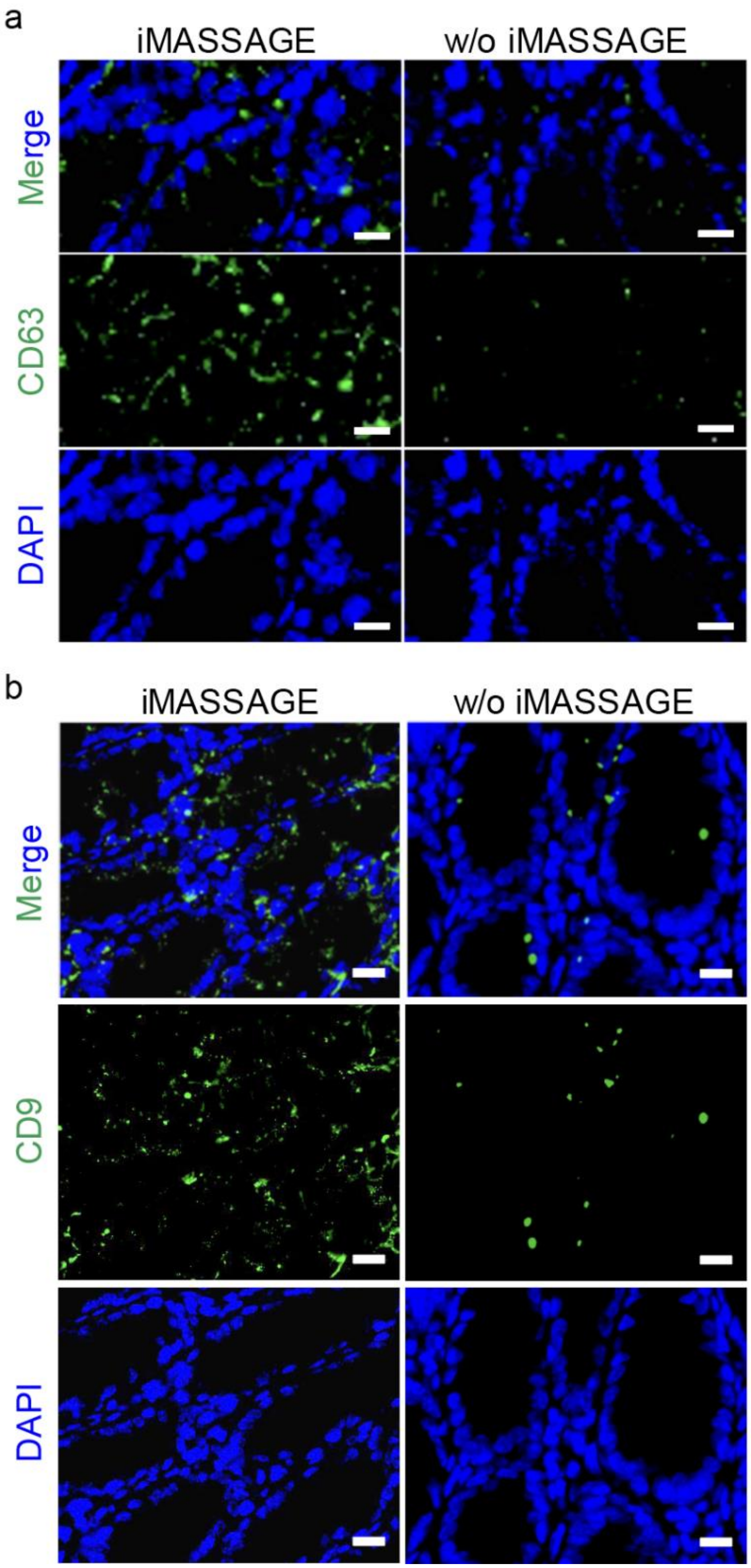

Supplementary Fig. 33. (a) CD63 and (b) CD9 immunofluorescent (green) at colonic tissue with or without iMASSAGE treatment. Scale bar: 10 μm.

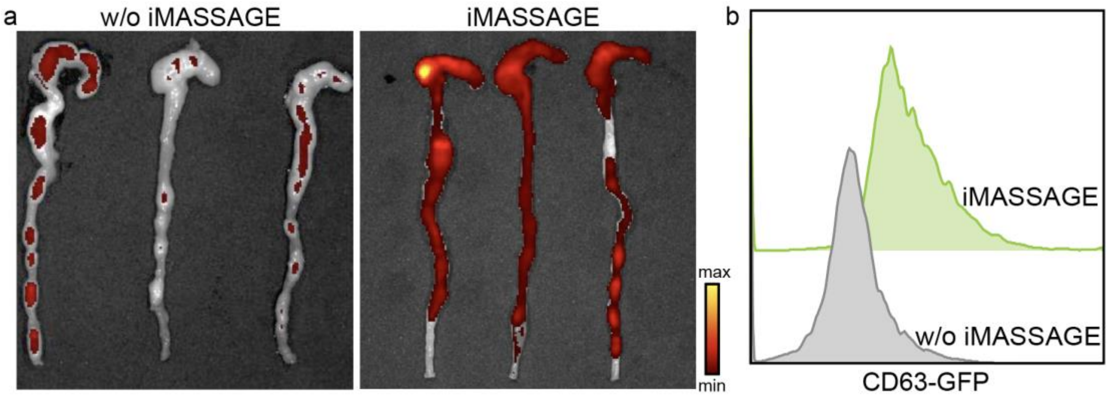

Supplementary Fig. 34. Detection of EVs produced by iMASSAGE device with stably transduced macrophage colonization in the intestinal lumen. (a) Fluorescent images of ex vivo colon in the group treated with pulsed wireless (1W, 3T). Ex: 488 nm, Em: 509 nm. (b) Flow cytometry analysis of EV containing CD63-GFP protein concentration in intestinal lumen contents after pulsed wireless stimulation (1W, 3T).

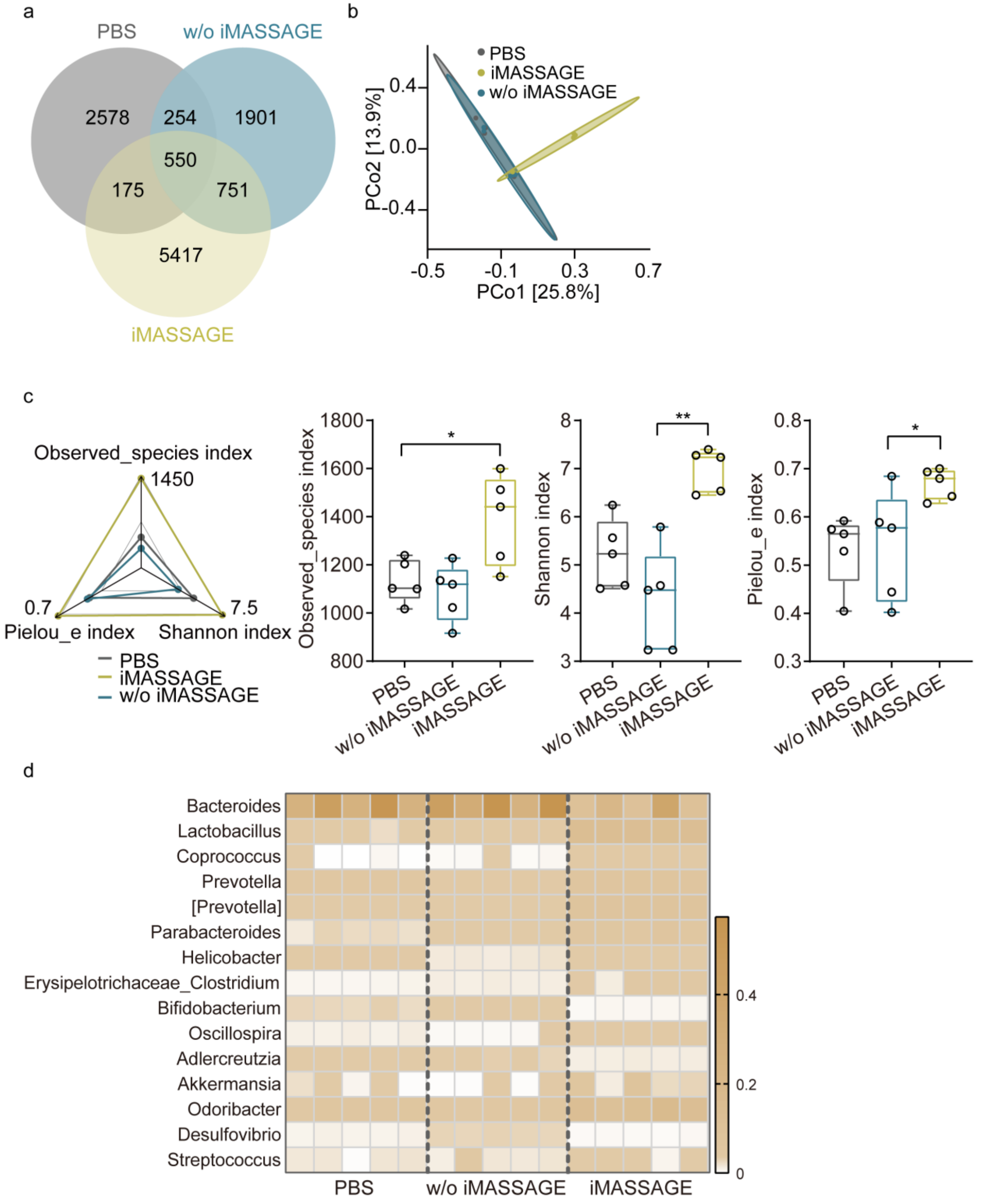

Supplementary Fig. 35. iMASSAGE-medicated regulation of the microbiota in mice with enteritis. (a) Venn diagram of all bacterial strains identified from differently treated mouse feces. (b) Principal coordinate analysis (PCoA) of the

Bray-Curtis distance based on OTUs after different treatments. (c) Radar plot and value of bserved_species index, pielou_e index and Shannon index of gut microbiota from mice feces for assessing the α diversity of the flora after different treatments (n=5 mice per group). Minima: Lower limit of the whisker; Maxima: Upper limit of the whisker; Centre: Median line inside the box; The upper and lower box bounds represent the 25% and 75% percentile of data. (d) Heatmap of the abundance of the top 15 microorganisms at the genus level. All data are presented as mean ± s.d.. The significant differences were calculated based on a two-tailed Student's t-test. **$p < 0.01$, *$p < 0.05$. Source data are provided as a Source Data file.

.

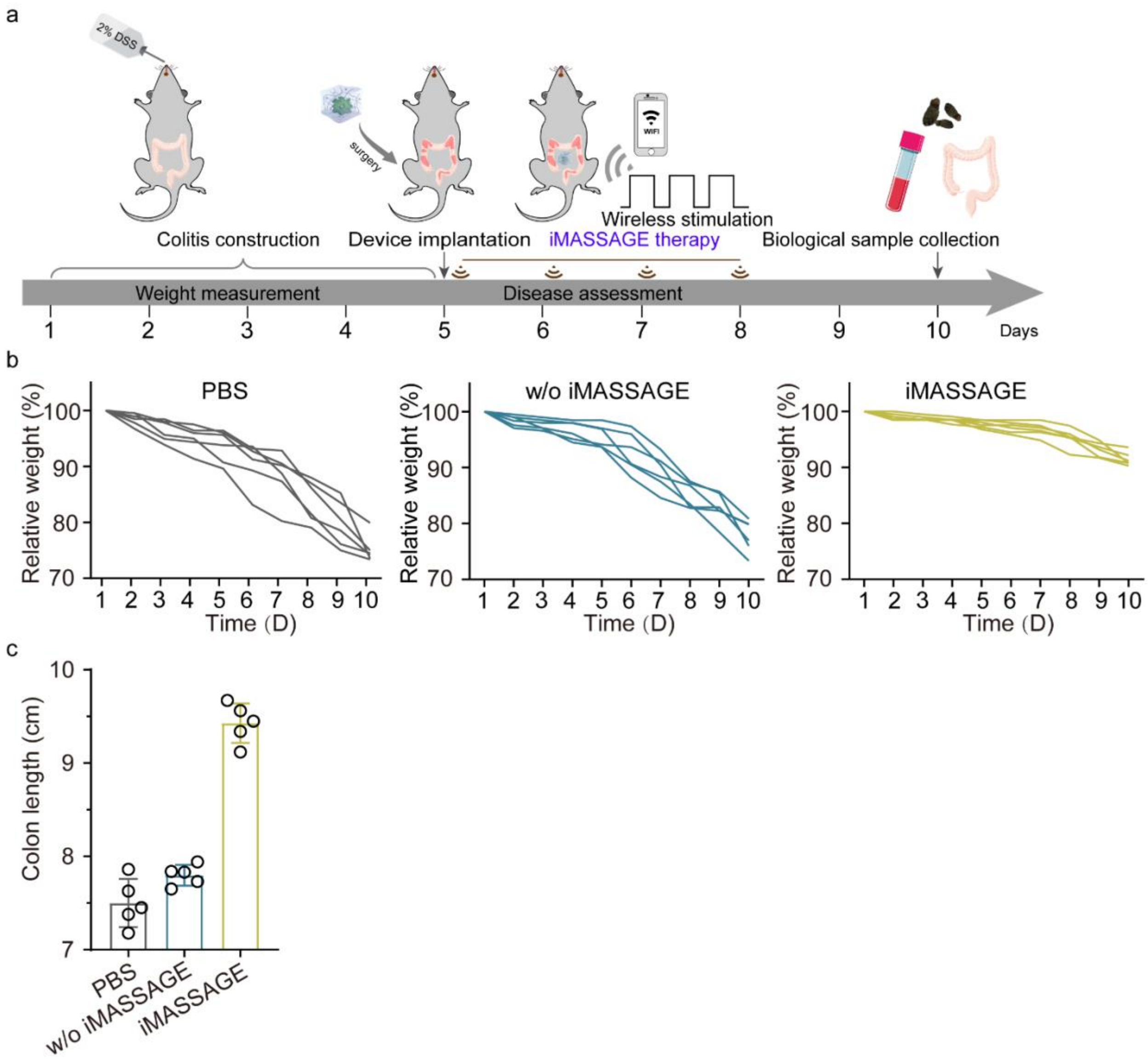

Supplementary Fig. 36. Implanted iMASSAGE device for IBD treatment. (a) Timeline of iMASSAGE-medicated colitis treatment by gut microbial modulation. After the colitis model was established by feeding water containing 2% DSS, the colitis mice were subjected to iMASSAGE therapy by receiving pulsed wireless stimulation (3 times/day), followed by collecting biological samples for further biological analysis. (b) Weight changes over time for each mouse in different group. (c) The length of colon ex vivo after different treatments (n=5 mice per group). All data are presented as mean ± s.d.. Source data are provided as a Source Data file.

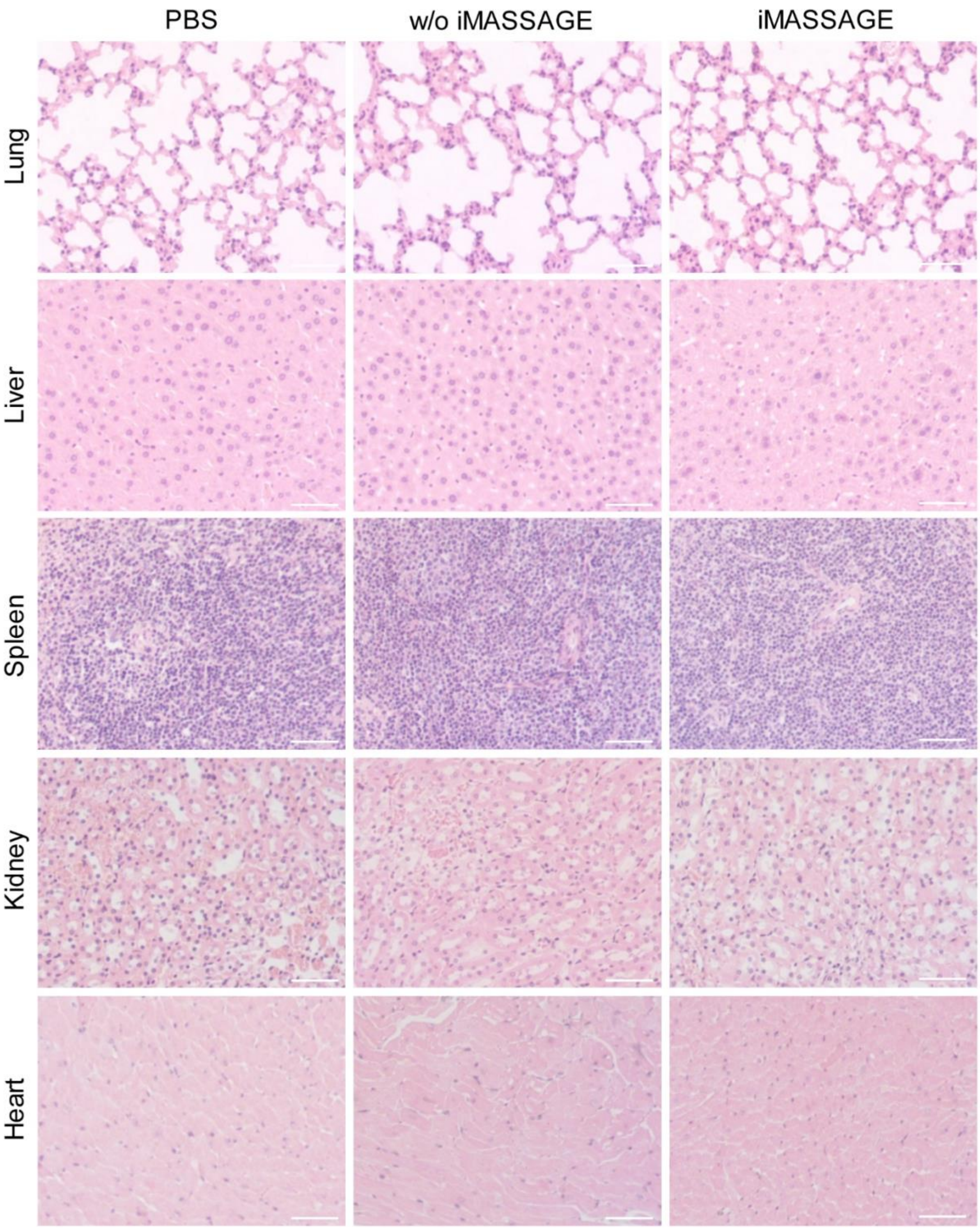

Supplementary Fig. 37. H&E tissue sections of major organs. Scale bar: 50 μm.

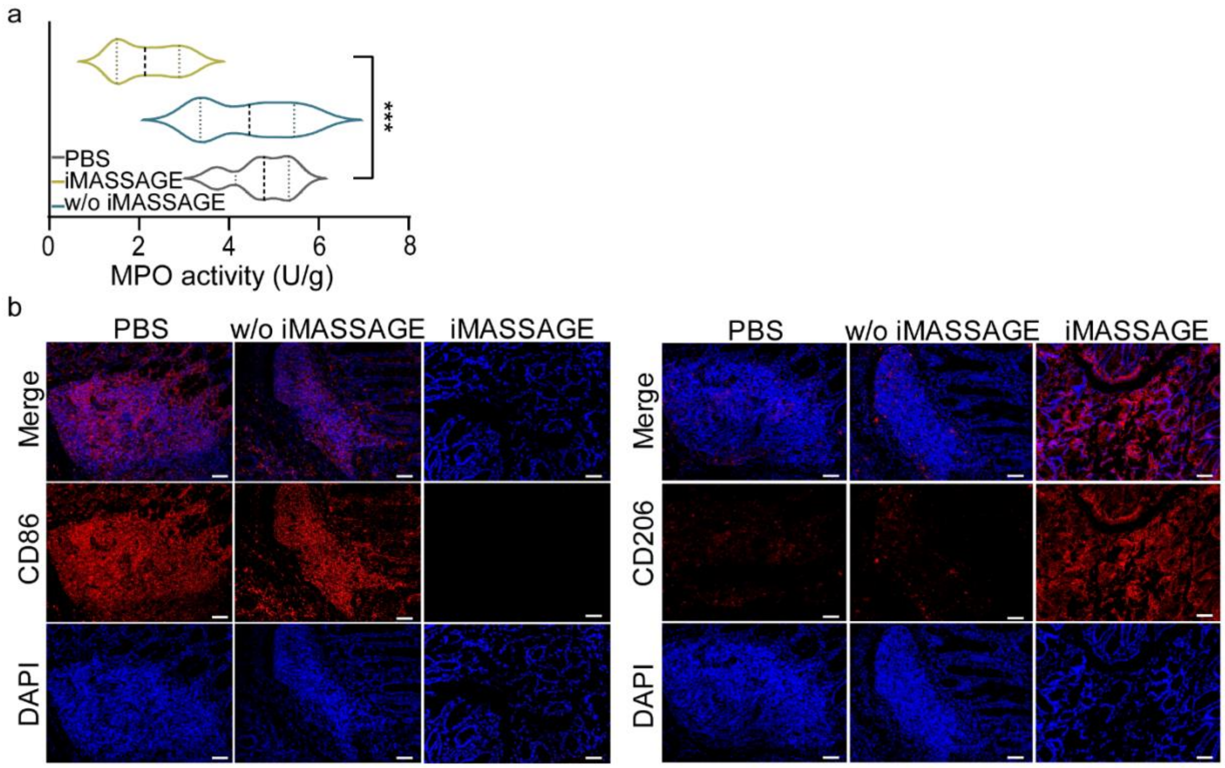

Supplementary Fig. 38. Infiltration of inflammatory cells in the colon after iMASSAGE treatment. (a) The MPO activity in colon tissue for assessing the infiltration of inflammatory neutrophils (n=5 mice per group). (b) Immunofluorescence images of macrophages in the colon of mice after different treatments. CD86 and CD206 served as indicators for pro-inflammatory M1 macrophages and anti-inflammatory M2 macrophages, respectively. Scale bar: 50 µm. The significant differences were calculated based on two-tailed Student's t-test. ***$p < 0.001$. All data are presented as mean ± s.d.. Source data are provided as a Source Data file.

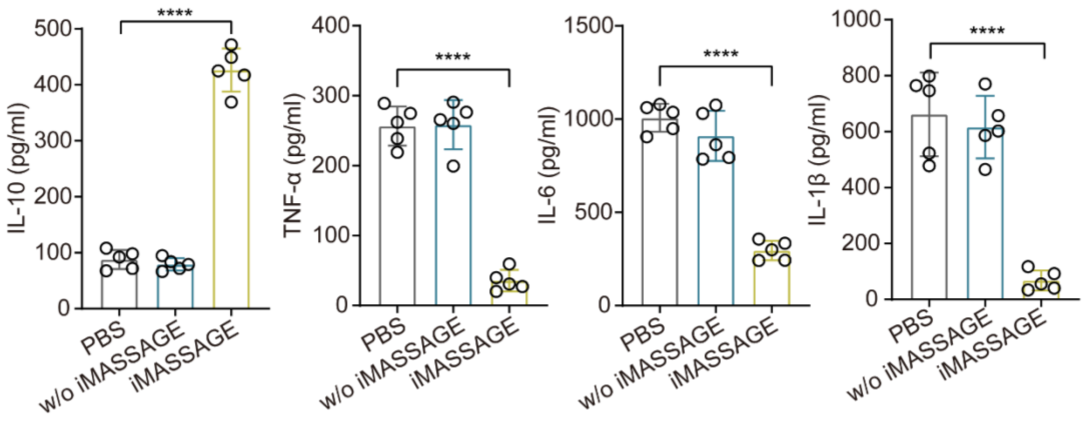

Supplementary Fig. 39. The expression of inflammatory factors in serum detected by ELISA kit (n=5 mice per group). The significant differences were calculated based on two-tailed Student's t-test. ****$p < 0.0001$. All data are presented as mean ± s.d.. Source data are provided as a Source Data file.

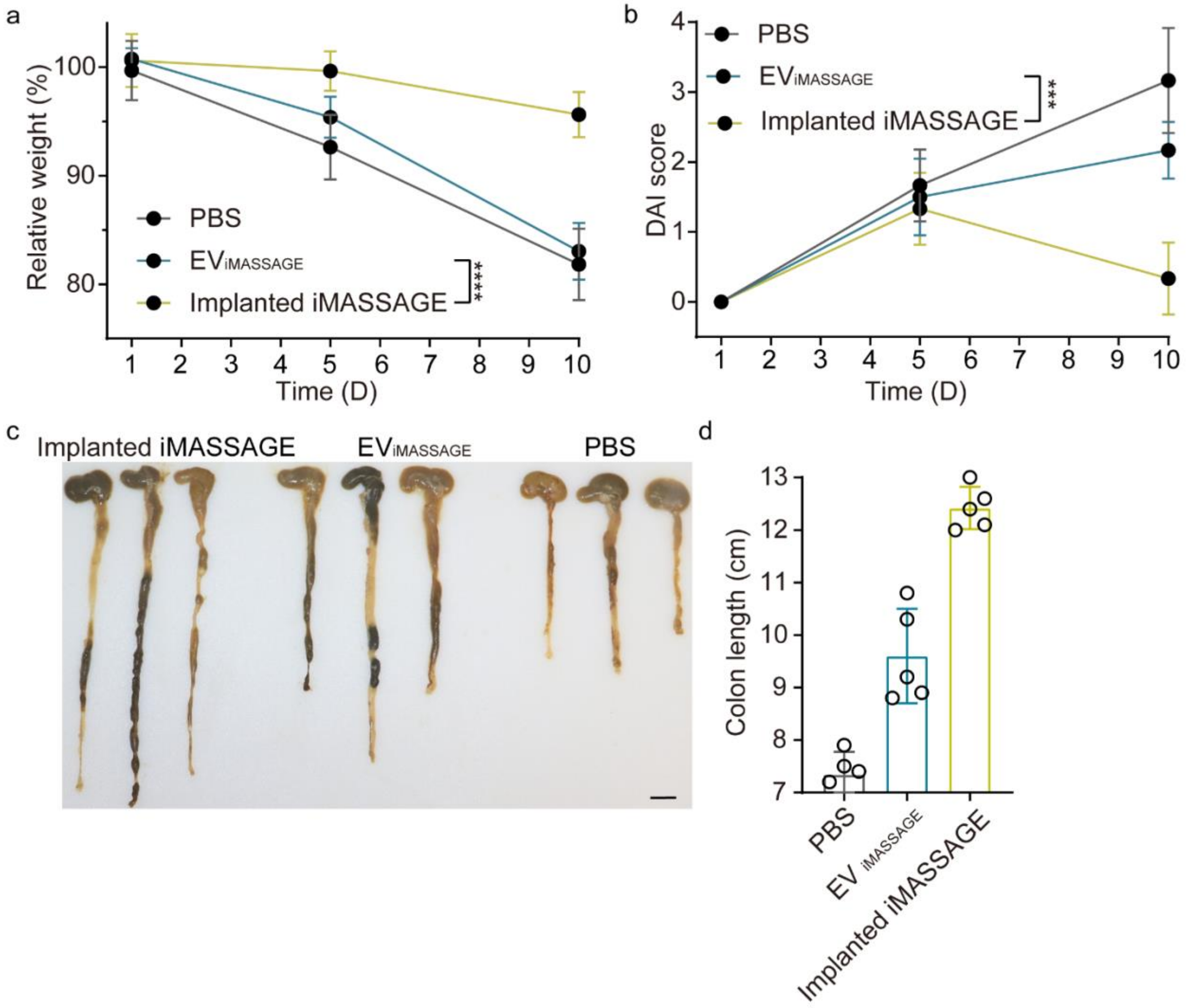

Supplementary Fig. 40. Therapeutic effect of IBD treatment with different administration methods. (a) Relative weight and (b) DAI score of mice after different treatments (n=5 mice per group). (c) Representative images and (d) length of isolated colon tissues ex vivo from different treated mice on day 10. Scale bar: 1cm. The significant differences were calculated based on two-tailed Student's t-test. ****$p < 0.0001$, ***$p < 0.001$. All data are presented as mean ± s.d.. Source data are provided as a Source Data file.

Supplementary Table 1. Information of used antibodies

Antibody	Provider	Application	Identifier
Anti-mouse CD63	Abcam	IF, WB	ab217345
Anti-mouse YAP1	Abcam	IF, WB	ab205270
Anti-mouse CD9	Cell Signal Technology	IF, WB	98327
Anti-mouse CD63	Biolegend	FC	143095
Anti-mouse CD11b	Biolegend	FC	101226
Anti-mouse CD80	Biolegend	FC	104708
Anti-mouse CD206	Biolegend	FC	141706
Anti-mouse CD206	Cell Signal Technology	IF	24595
Anti-mouse CD86	Cell Signal Technology	IF	76755
Anti-mouse F-actin	Abcam	WB	ab233267
Anti-mouse VPS4	Abcam	WB	ab181078
Anti-mouse GAPDH	Abcam	WB	ab181602
