# Supplementary material for: Mechanoelectronic stimulation of autologous extracellular vesicle biosynthesis implant for gut microbiota modulation
Source: Nat Commun. 2024 Apr 18;15:3343. doi: 10.1038/s41467-024-47710-w (PMC11026491; doi:10.1038/s41467-024-47710-w)
Supplement: Supplementary file 3 — Reporting Summary [file 41467_2024_47710_MOESM3_ESM.pdf]

nature portfolio

Corresponding author(s): Xianguang Ding, David Tai Leong, Lianhui Wang

Last updated by author(s): 2024-3-23

Reporting Summary

Nature Portfolio wishes to improve the reproducibility of the work that we publish. This form provides structure for consistency and transparency in reporting. For further information on Nature Portfolio policies, see our Editorial Policies and the Editorial Policy Checklist.

Statistics

For all statistical analyses, confirm that the following items are present in the figure legend, table legend, main text, or Methods section.

n/a	Confirmed	
☐	☒	The exact sample size (*n*) for each experimental group/condition, given as a discrete number and unit of measurement
☐	☒	A statement on whether measurements were taken from distinct samples or whether the same sample was measured repeatedly
☐	☒	The statistical test(s) used AND whether they are one- or two-sided<br>*Only common tests should be described solely by name; describe more complex techniques in the Methods section.*
☒	☐	A description of all covariates tested
☒	☐	A description of any assumptions or corrections, such as tests of normality and adjustment for multiple comparisons
☐	☒	A full description of the statistical parameters including central tendency (e.g. means) or other basic estimates (e.g. regression coefficient) AND variation (e.g. standard deviation) or associated estimates of uncertainty (e.g. confidence intervals)
☐	☒	For null hypothesis testing, the test statistic (e.g. *F*, *t*, *r*) with confidence intervals, effect sizes, degrees of freedom and *P* value noted<br>*Give P values as exact values whenever suitable.*
☒	☐	For Bayesian analysis, information on the choice of priors and Markov chain Monte Carlo settings
☒	☐	For hierarchical and complex designs, identification of the appropriate level for tests and full reporting of outcomes
☒	☐	Estimates of effect sizes (e.g. Cohen's *d*, Pearson's *r*), indicating how they were calculated

Our web collection on statistics for biologists contains articles on many of the points above.

Software and code

Policy information about availability of computer code

Data collection	Laser scanning confocal microscope (Leica TCS SP8, Germany), flow cytometry (BD Accuri C6), multi-plate reader (Infinite 200 PRO, Switzerland), scanning electron microscopy (S4800), electromechanical universal testing machine (CMT6502), Computed Tomography (Nano Voxel 1-2702E).
Data analysis	General data were analyzed by graphpad prism 8 and origin 2022.

For manuscripts utilizing custom algorithms or software that are central to the research but not yet described in published literature, software must be made available to editors and reviewers. We strongly encourage code deposition in a community repository (e.g. GitHub). See the Nature Portfolio guidelines for submitting code & software for further information.

Data

Policy information about availability of data

All manuscripts must include a data availability statement. This statement should provide the following information, where applicable:

- Accession codes, unique identifiers, or web links for publicly available datasets
- A description of any restrictions on data availability
- For clinical datasets or third party data, please ensure that the statement adheres to our policy

All data are available in the main text or the supplementary materials. Source data are provided with this paper.

Research involving human participants, their data, or biological material

Policy information about studies with human participants or human data. See also policy information about sex, gender (identity/presentation), and sexual orientation and race, ethnicity and racism.

Reporting on sex and gender	N/A
Reporting on race, ethnicity, or other socially relevant groupings	N/A
Population characteristics	N/A
Recruitment	N/A
Ethics oversight	N/A

Note that full information on the approval of the study protocol must also be provided in the manuscript.

Field-specific reporting

Please select the one below that is the best fit for your research. If you are not sure, read the appropriate sections before making your selection.

☒ Life sciences ☐ Behavioural & social sciences ☐ Ecological, evolutionary & environmental sciences

For a reference copy of the document with all sections, see nature.com/documents/nr-reporting-summary-flat.pdf

Life sciences study design

All studies must disclose on these points even when the disclosure is negative.

Sample size	Sample size were determined based on the volume of experimental mice.
Data exclusions	No data was excluded
Replication	Each experiment was repeated at least three times
Randomization	All samples were randomly allocated into experimental groups.
Blinding	Except for device construction, all other experiments were blinded to group allocation during data collection and analysis.

Reporting for specific materials, systems and methods

We require information from authors about some types of materials, experimental systems and methods used in many studies. Here, indicate whether each material, system or method listed is relevant to your study. If you are not sure if a list item applies to your research, read the appropriate section before selecting a response.

Materials & experimental systems

n/a	Involved in the study	
☐	☒	Antibodies
☐	☒	Eukaryotic cell lines
☒	☐	Palaeontology and archaeology
☐	☒	Animals and other organisms
☒	☐	Clinical data
☒	☐	Dual use research of concern
☒	☐	Plants

Methods

n/a	Involved in the study	
☒	☐	ChIP-seq
☐	☒	Flow cytometry
☒	☐	MRI-based neuroimaging

Antibodies

Antibodies used	Anti-mouse CD63 (ab217345), anti-mouse YAP1 (ab205270), anti-mouse F-actin (ab233267), anti-mouse VPS4 (ab181078) and anti-mouse GAPDH (ab181602) were purchased from Abcam. Anti-mouse CD63 (143095), Anti-mouse CD11b (101226), anti-mouse CD80 (104708) and anti-mouse CD206 (141706) were purchased from Biolegend. Anti-mouse CD206 (24595), anti-mouse CD9 (98327) and

April 2023

	anti-mouse CD86 (76755) were purchased from Cell Signal Technology.
Validation	https://www.abcam.com/<br>https://www.cellsignal.com/<br>https://www.biolegend.com/

Eukaryotic cell lines

Policy information about cell lines and Sex and Gender in Research

Cell line source(s)	All cell lines used in the experiments were purchased from ATCC.
Authentication	The cell lines were authenticated using a short tandem repeat DNA profiling method and results were compared with database.
Mycoplasma contamination	All cell lines tested negative for mycoplasma contamination by a standard PCR protocols
Commonly misidentified lines (See ICLAC register)	No commonly misidentified cell lines were used.

Animals and other research organisms

Policy information about studies involving animals; ARRIVE guidelines recommended for reporting animal research, and Sex and Gender in Research

Laboratory animals	BALB/c mice, femal, 6-8 weeks. All mice were fed at a temperature of 18-22 degree and a humidity of 50%-60%.
Wild animals	No wild animals were used.
Reporting on sex	Female mice were used for the in vivo experiments in this study.
Field-collected samples	No field-collected samples were used.
Ethics oversight	All Animal experiments were carried out in accordance with ethical guidelines and approved by the Committee for Animal Research of Nanjing University of Posts and Telecommunications (No: 202202).

Note that full information on the approval of the study protocol must also be provided in the manuscript.

Plants

Seed stocks	N/A
Novel plant genotypes	N/A
Authentication	N/A

Flow Cytometry

Plots

Confirm that:

☒ The axis labels state the marker and fluorochrome used (e.g. CD4-FITC).

☒ The axis scales are clearly visible. Include numbers along axes only for bottom left plot of group (a 'group' is an analysis of identical markers).

☒ All plots are contour plots with outliers or pseudocolor plots.

☒ A numerical value for number of cells or percentage (with statistics) is provided.

Methodology

Sample preparation	EVs derived from macrophages (RAW264.7, ATCC: TIB-71) and human intestinal epithelial cells (ATCC: HIEC-6) were collected by gradient centrifugation. Briefly, cell culture supernatants were centrifuged at 2,000 g for 10 min, followed by

centrifugation at 10,000 g for 30 min. The final EVs were obtained by collecting the pellet after centrifugation (100,000g, 2 h). DiD-labeled EVs (20 µg/mL, 200 µL) were co-cultured with Calcein- AM-stained gut microbiota (107 CFU/mL), including Staphylococcus aureus (ATCC 23235, S. aureus,) and Escherichia coli (ATCC 25922, E. coli) for 4 h, respectively. After washing three times with PBS (8,000 rpm, 10 min), the uptake of EVs by microbiota was observed using a laser scanning confocal microscope (Leica TCS SP8, Germany) and analyzed by flow cytometry (BD Accuri C6).

Instrument	Flow cytometry (BD Accuri C6)
Software	FlowJo v10.8.1
Cell population abundance	10^9-10^10 cells/mL
Gating strategy	Cell were gated based on size and granularity of forward and side scatter and further analyzed for specific fluorescence.

☒ Tick this box to confirm that a figure exemplifying the gating strategy is provided in the Supplementary Information.
